# Gathering, processing, and interpreting information about COVID-19

**DOI:** 10.1038/s41598-021-86088-3

**Published:** 2021-03-22

**Authors:** Arnout B. Boot, Anita Eerland, Joran Jongerling, Peter P. J. L. Verkoeijen, Rolf A. Zwaan

**Affiliations:** 1 Department of Psychology, Education, and Child Studies, Erasmus University Rotterdam, Mandeville Building, Room T13-44, Burgemeester Oudlaan 50, 3062 PA Rotterdam, The Netherlands; 2 Department of Languages, Literature, and Communication, Utrecht University, Utrecht, The Netherlands; 3 Brain and Learning Research Group, Learning and Innovation Center, Avans University of Applied Sciences, Breda, The Netherlands

**Keywords:** Psychology, Human behaviour

## Abstract

Does cognitive motivation influence how people gather and interpret information about COVID-19 and their adherence to measures? To address these questions, we conducted a longitudinal survey among European and American respondents. Wave 1 (*N* = 501) was conducted on March 27, 2020 and Wave 2 (*N* = 326) on July 1, 2020. We assessed COVID-19 knowledge, endorsement of COVID-19 conspiracy theories, media use, Need for Cognition (NC), Need for Cognitive Closure (NCC), and self-reported adherence to governmental measures taken. Results showed that nearly three-quarters of our respondents actively searched for information about COVID-19. Most at least once a day. Information seeking behaviour was not influenced by cognitive motivation (i.e., NC and NCC). However, cognitive motivation was related to (1) knowledge about COVID-19, (2) conspiracy rejection, and (3) change in knowledge over time. Respondents with more knowledge on COVID-19 also indicated to adhere more often to measures taken by their government. Self-reported adherence to measures was not influenced by cognitive motivation. Implications of these findings will be discussed.

## Introduction

A world-altering event provides a shock to the systems by which we, and others around us, try to make sense of our environment. Since the advent of the digital media, major events are typically followed by a daily barrage of information, misinformation, and disinformation. In this study, we investigated whether people’s cognitive motivation to understand the world could predict how people acquire, interpret, and process information about COVID-19. Specifically, what is the extent of factual knowledge people have about COVID-19? How stable across time is this knowledge? How frequently do they search for new information? Which information sources do they rely on? How confident are they about what they know? How do they evaluate false conspiracy theories? And how is their knowledge related to their behaviour? We investigated these questions in a survey that examined people's responses to COVID-19, by many seen as the most impactful global event since World War II.

COVID-19, the infectious disease caused by severe acute respiratory syndrome coronavirus 2, has resulted in a worldwide pandemic (declared by the World Health Organization on March 11, 2020). As individuals in many countries were practicing social distancing in the early spring of 2020, they were largely reliant on the (digital) media to gather information and develop an understanding of developments related to COVID-19. As of April 7, 2020, fact checkers have identified 225 pieces of misinformation about COVID-19^1^. The most common claims within pieces of misinformation concerned the actions or policies that public authorities were taking to address COVID-19.

To examine people’s gathering and comprehension of information related to the COVID-10 pandemic, we conducted an online survey in two waves. The first wave was conducted on March 27 of 2020 among 501 adults who are fluent in English while the second wave was conducted on July 1 of 2020 among 326 adults that also participated in Wave 1. We asked about people’s (digital) media use and their knowledge and beliefs about COVID-19. In Wave 1 we administered two standardized tests of cognitive motivation, Need for Cognition^2–4^, and Need for Cognitive Closure^5^. We used these measures to assess responses in Wave 1 as well as any changes on the COVID-19 Knowledge Test responses in Wave 2. As will be detailed below, each wave was preregistered separately.

### Need for cognition

To presuppose specific COVID-19 information processes we considered two distinct types of cognitive motivations. Our first hypothesis was based on the Need for Cognition (NC), a scale that reflects the pleasure one experiences in one’s own thought processes^2,4^. People who are high in NC experience pleasure from engaging in effortful cognitive processes, whereas people low in NC people are less likely to enjoy these processes^4^. High NC people also tend to be more resistant to persuasive messages by performing a more effortful analysis and cognitive reflection on the quality of the information^6^. Based on these previous findings, we expected that a person high in NC would be likely to seek out a great deal of information about COVID-19, consider all the pertinent information by consulting a broad variety of resources, and would think deeply and critically about the topic. Therefore, we also expected people high in NC to acquire a substantial amount of factual knowledge about the virus.

Furthermore, we assumed that it is more probable that conventional media (newspapers, radio, TV) can endure close scrutiny than social media. For example, conventional media are often based on traditional journalism and (arguably) more objective news reports, whereas social media often comprise tabloid journalism and unofficial sources, which tend to convey more sensation-driven news stories and subjective discourse, respectively^7^. Additionally, social media users have been shown to spread false news more quickly and more broadly than true news^8^. Thus, we hypothesized that if a person high in NC is more likely to focus on objective and factual news about COVID-19 than on subjective discourse on this topic, this person would be more likely to use conventional media.

#### Hypothesis 1

(a) People who have relatively low NC (vs. high NC) have less factual knowledge about COVID-19—(b) they show a lower frequency of COVID-19 information updates—(c) and they rely more on new media and informal sources (e.g., social media, colloquial conversations) to acquire new information about COVID-19.

### Need for cognitive closure

We based our second hypothesis about cognitive motivation and COVID-19 information processing on the Need for Cognitive Closure (NCC). Where NC focuses on the process of thinking, NCC focuses on the outcome of the thought process. Individuals who are high in NCC are motivated to quickly arrive at an interpretation of a state of affairs and then preserve this interpretation in the face of incoming information. These two intellectual moves are known as *seizing* and *freezing*, respectively^9,10^. NCC is both situational (i.e., people experience it to a greater degree in urgent situations than in less urgent situations) and dispositional (i.e., some people experience it more than others). We focus on the dispositional component of NCC in the current study, given that we are primarily interested in individual differences. Also, we expected people to experience a great deal of NCC during the early phase of a pandemic. Individuals low in NCC are thought to be less eager to arrive at and hold on to an interpretation of a state of affairs. Presumably, this means people low in NCC are more likely to hedge their answers when their knowledge is being tested. For example, a person low in NCC could be more likely to judge a true statement with ‘I think this is true’, whereas a person high in NCC could be more likely to answer with ‘I am sure this is true’.

#### Hypothesis 2

People high in NCC have more confidence in their acquired knowledge than people low in NCC.

### Proneness to conspiracy beliefs

We are also interested in the degree to which individuals are willing to endorse one or more conspiracy-related assumptions about COVID-19. Conspiracy theories provide readily available answers to convoluted issues such as the pandemic, and can therefore mitigate feelings of uncertainty^11^. Thus, one might expect that high NCC individuals, who wish to diminish uncertainties (i.e., cognitive closure), would be more likely to endorse conspiracy theories about the pandemic. Still, several studies report at best weak correlations between the two^12–14^. However, it has been observed that these studies examined the link between NCC and conspiracy beliefs in contexts in which conspiracy theories would be particularly inaccessible to the individual^11^. Indeed, when conspiracy theories were made more accessible to respondents, a correlation between NCC and conspiracy thinking emerged. Moreover, when conspiracy theories are situationally accessible explanations for real events, people high in NCC are more likely to accept these conspiracy theories as truths^11^. Considering the COVID-19 infodemic, it is very likely that social media users have been exposed to conspiratorial explanations for the pandemic at least occasionally. For example, it is been shown that Twitter posts related to COVID-19 have a questionable to reliable source ratio of 0.11, which means there are approximately 11 unreliable posts for every 100 reliable posts on Twitter^15^. Likewise, YouTube suffers a ratio of 0.07, meaning there are 7 questionable videos posted about COVID-19 for every 100 reliable videos^15^. Thus, if high NCC can predict a higher proneness to conspiracy beliefs under the prerequisite that these theories are situationally accessible, then we expect high NCC individuals who use less reliable sources such as social media (which is presumably predicted by low NC) to be particularly susceptible to adopt conspiracy beliefs. Therefore, our third hypothesis derives from a potential interaction between NC and NCC. All hypotheses of this study are summarized in Table 1.

**Table 1:** Hypotheses regarding COVID-19 Knowledge, conspiracy rejection, and media use based on need for cognition and need for cognitive closure.

	Need for cognition (NC)		Hypotheses
	Low*	High*	**
**Need for cognitive closure (NCC)**			
Low*	Low COVID-19 knowledge	High COVID-19 knowledge	1a
	Low searching	High searching	1b
	Low quality of sources	High quality of sources	1c
	Low certainty	Low certainty	2
	High rejection of conspiracy theories***	High rejection of conspiracy theories	3
High*	Low COVID-19 knowledge	High COVID-19 knowledge	1a
	Low searching	High searching	1b
	Low quality of sources	High quality of sources	1c
	High certainty	High certainty	2
	Low rejection of conspiracy theories	High rejection of conspiracy theories	3

*Previous studies have categorized NC and NCC scales in ‘high’ and ‘low’ groups, thus dichotomizing continuous scores in statistical analysis 7,13–16. However, categorization is unnecessary for statistical analysis and even has methodological weaknesses 17. That said, labelling participants as high or low groups does allow for a convenient comparison of the outcome variables. To achieve the middle ground, we have chosen to use continuous variables in our statistical analyses, whereas in our descriptive statistics we have added comparisons based on high and low NC–NCC categories.

**Hypotheses 1a-c are based on NC, Hypothesis 2 is based on NCC, and hypothesis 3 is based on an interaction between NC and NCC.

***In our preregistration hypothesis 3 was formulated in terms of ‘endorsement’ instead of ‘rejection’.

#### Hypothesis 3

People who are high in NCC but low in NC are less likely to reject conspiracy theories about COVID-19 than high NCC & high NC people, low NCC & high NC people, and low NCC & low NC people.

### Behaviour in the pandemic and temporal changes in knowledge

In addition to factual knowledge about COVID-19, another important aspect of the pandemic is behaviour. Particularly, the effectiveness of health-regulations and guidelines against the coronavirus is dependent on the level of compliance and adherence amongst the population. To examine this idea, we added two additional questions in Wave 2 about adherence behaviour and the perceived importance of different organizations such as one’s government or the World Health Organization (WHO). Furthermore, we considered it valuable to perform a second iteration of the knowledge measures to study temporal differences. Thus, complementary to the Wave 1 analysis, we used an exploratory approach to analyse Wave 2 data and asked whether cognitive motivation (NC and NCC), the guiding principle in this study, could predict adherence behaviour as well as temporal changes in COVID-19 knowledge between Wave 1 (March 27) and Wave 2 (July 1).

## Wave 1

### Method

We administered two surveys: the first survey (Wave 1) was administered on March 27 of 2020, and on July 1 of 2020 we sent out a second survey (Wave 2). The first survey was designed to assess the level of knowledge about COVID-19, the ability to reject COVID-19-related conspiracy theories, cognitive motivations, media use related to COVID-19, general media use, and demographic traits (see Table 2 for more detailed descriptions). Wave 2 was intended to gather additional data about potential temporal changes in COVID-19 Knowledge, Conspiracy Rejection, as well as additional information about adherence to government-imposed measures (described in more detail in the Wave 2 section). Wave 1 and Wave 2 data were analysed separately. The current section (i.e., Wave 1) proceeds with Wave 1 methods and analyses.

**Table 2:** Overview and descriptions of the measures in wave 1.

Category	Measure	Items	Description
Information processing	COVID-19 Knowledge	24	*Knowledge about COVID-19* in terms of accuracy and confidence
	Conspiracy Rejection	8	*COVID-19-related conspiracy rejections* in terms of accuracy and confidence
Cognitive motivation	Need for Cognition Scale (NC)^2^	18	The desire to engage in cognitive activities (e.g., critical thinking, acquiring a deeper understanding of certain topics)
	Need for Cognitive Closure Scale (NCC)^16^	47	The desire for coherence in personal thought processes. High NCC predicts a higher likelihood to arrive more quickly at interpretations and more definitive inferences about the situation (i.e., ideationally crystalized worldview). Low NCC predicts more cognitive flexibility and higher acceptance to uncertainties (i.e., ideationally fluid worldview)
COVID-19 media use	Sources	10	The extent to which different types of sources are used to acquire information about COVID-19 (e.g., news sites, social networking sites, friends/family)
	Conditions	4	The conditions of COVID-19 news encounters (i.e., active searching, coincidentally, colloquially, and avoidance behaviour)
	Frequency	1	The frequency of COVID-19 information updates
	Motivations	5	The underlying motivations to acquire information about COVID-19 (i.e., need to be informed, need to educate others, concerns about personal health, relatives, and the general public)
General media use	Platforms used	1	The online platforms that are used by the participant (i.e., online media environment)
	Daily time spent	1	The average daily time spent on the online platforms
Demographic traits	Various measures	7	Age, gender identity, career field, nationality, native language, and level of education

#### Respondents

Five hundred and twenty-six respondents completed the Wave 1 survey via the Prolific.com platform. Sixteen respondents were excluded for spending too much time on the survey. Another nine respondents were excluded for failing the catch question. In our preregistration we included an additional exclusion criterion based on the lie-score of the respondents on the NCC^16^. In hindsight, however, this criterion proved to be undesirable: the data strongly suggested that the lie score was not a valid measurement for respondents’ mischief. That is, respondents with a lie score > 15 still passed the catch-question and commented on the survey in an ostensibly proactive and honest manner. Moreover, we believe that the answers in the “lie” questions were partially influenced by a social-desirability bias and were prone to idiosyncratic interpretations of the adverbs in the response options. For example, the item *“I have never been late for an appointment or work.”* was answered with “*slightly agree*”, “*moderately agree*”, and “*strongly agree*” by 10.2, 20.0, and 12.8 percent of the respondents, respectively. According to the initial lie-item exclusion criterion, 43 percent of respondents gave a response that would indicate lying, which appears highly unlikely. A final reason for the decision to remove the lie-score criterion was the questionable Cronbach's α of 0.62.

The final sample included 501 respondents (238 females). Respondents’ ages ranged from 18 to 77 (*M* = 31.25, *SD* = 11.33). See Supplementary Tables 1–5 in online Appendix A at https://osf.io/kw9hy/ for descriptive statistics regarding the career fields, level of education, nationality, native language, and gender identity of our Prolific sample. On average, the survey took 22 min to complete. Respondents were paid £2.75, amounting to an average hourly fee of £7.50. All respondents were fluent in English according to the Prolific database.

#### Materials and procedure

Respondents first filled out the COVID-19 Knowledge Test that we developed for this survey. This test consists of 32 statements regarding the state of knowledge about COVID-19 as it was in March 2020. Twelve statements were thought to be correct (e.g., *Fever is a symptom of the coronavirus*), twelve were thought to be incorrect (e.g., *Flu vaccine protects you against the coronavirus*), and eight concerned inferred motives (e.g., *The coronavirus was released by the Chinese government to prevent overpopulation*). We expected the twelve correct and incorrect statements to measure individuals’ knowledge of COVID-19, while the eight items on inferred motives were thought to measure individuals’ belief in conspiracy theories regarding the virus. Respondents indicated whether or not they thought these statements were true, and how confident they felt about their judgment (1 = *I am sure this is not true*, 2 = *I think this is not true*, 3 = *I don’t know*, 4 = *I think this is true*, 5 = *I am sure this is true*). In the current study, 'COVID-19 Knowledge' was defined as 'scientifically justified belief on an ordinal spectrum of accuracy weighted by confidence level'. After reverse-coding the false statements we interpreted the five-point scale accordingly (1 = *highly inaccurate*, 2 = *inaccurate*, 3 = *neutral*, 4 = *accurate*, 5 = *highly accurate*). In addition, respondents filled out questions about media use related to COVID-19, general media use, and demographic traits (see Table 2).

The measures of Wave 1 reflect cognitive motivations (NC and NCC), information processing and interpretation (COVID-19 Knowledge and Conspiracy Rejection) and information seeking behaviour (sources, conditions of news encounters, frequency, and motivations).

### Availability of materials and data

The data, preregistration, and materials from this study are available here https://osf.io/38b65/files/. The data can be converted to other file types (currently R format) and the R syntax used in this study can be made available upon request.

### Ethical approval

This study has been approved by the Ethics review Committee DPECS (EC-DPECS), Erasmus University Rotterdam. This study was performed in accordance with guidelines and regulations.

### Informed consent

All respondents provided their informed consent to participate in this study.

## Results

The results are presented in two parts. First, we present our confirmatory factor, regression, and cluster analyses. Next, we present descriptive statistics regarding the COVID-19 Knowledge and Conspiracy items and COVID-19-related media use.

### Confirmatory analyses

We tested whether the COVID-19 Knowledge Test consisted of two different factors (COVID-19 *Knowledge* and *Conspiracy Rejection*). To this end, we ran a confirmatory factor analysis in the R-package *lavaan*^17^. Specifically, we fitted a two factor model using robust weighted least square estimation. The first factor, *Knowledge*, was the accuracy score for the first 24 items of the COVID-19 Knowledge test. The second factor, *Conspiracy Rejection*, was the accuracy score on the last eight items of the COVID-19 Knowledge test. This factor-structure was fit to a random subsample of 200 individuals, and validated on the remaining respondents in our sample.

We removed items 4, 5, 17, 29, and 31 from our analyses, because some answer options for these ordinal variables were not present in our subsample. After removing these items, the two-factor model showed sufficient fit to the data (χ^2^(323) = 399.52, *p* = 0.002; CFI = 0.988; RMSEA = 0.035; SRMR = 0.086). When validating the model on the remaining 301 respondents, we removed item 16 and 25 due to unused answer options. In our validation sample items 4, 5, 17, 29, and 31 were not removed. Model fit for the 2-factor model was again sufficient (χ^2^(404) = 637.80, *p* < 0.001; CFI = 0.977; RMSEA = 0.046; SRMR = 0.085). When fitted to the whole sample, no items needed to be removed and the model showed good fit (χ^2^(433) = 781.06, *p* < 0.001; CFI = 0.985; RMSEA = 0.041; SRMR = 0.071). These results indicate that COVID-19 *Knowledge* and *Conspiracy Rejection* can be distinguished as factors in the COVID-19 Knowledge Test.

### Test of NC and NCC as predictors

We were interested in whether NC and NCC predict the COVID-19 Knowledge Test results and COVID-19-related information searching frequency. Therefore, we chose to perform a regression analysis. Adding NC, NCC, and their interaction as predictors for the *COVID-19 Knowledge* and *Conspiracy Rejection* factors lead to a model that also has sufficient fit to the data (χ^2^(553) = 979.06 , *p* < 0.001; CFI = 0.981; RMSEA = 0.041; SRMR = 0.073). Results show that NC was positively related to *COVID-19 Knowledge* (b(se) = 0.254 (0.062), beta = 0.242, *p* < 0.001), which supported **hypothesis 1a** (i.e., *low NC is associated with less factual knowledge about COVID-19*). NC was also positively related to *Conspiracy Rejection* (b(se) = 0.136 (0.057), beta = 0.134, *p* < 0.001), high NC participants were more likely to reject false conspiracy theories about COVID-19. However, there were no significant relations between NC and media use at Wave 1 (NC: b(se) = 0.070 (0.052), beta = 0.070, *p* = 0.180; NCC: b(se) = 0.062 (0.053), beta = 0.062, *p* = 0.242). Therefore, there was no support for **hypothesis 1b** (i.e., *people who have relatively low NC show a lower frequency of COVID-19 information updates)*.

NCC, the other measure of cognitive motivation, was positively related to *COVID-19 Knowledge* (b(se) = 0.252 (0.061), beta = 0.240, *p* < 0.001), and was positively related to *Conspiracy Rejection* (b(se) = 0.143 (0.054), beta = 0.141, *p* = 0.008). This means high NCC predicted higher scores on the COVID-19 Knowledge Test. However, descriptive analyses of the data had to be used to examine whether scores could be caused by higher confidence. Thus, the results from the inferential analysis could not conclusively support or disconfirm **hypothesis 2** (i.e., *people high in NCC have more confidence in their acquired knowledge than people low in NCC*). This limitation will be further discussed.

In addition, there was a significant interaction of NC and NCC on *COVID-19 Knowledge* (b(se) = -0.109 (0.051), beta = -0.104, *p* = 0.032). Participants who were both high in NC and NCC showed higher levels of *COVID-19 Knowledge*. However, there was no significant NC x NCC interaction effect on *Conspiracy Rejection*. Therefore, the results did not support **hypothesis 3** (i.e., *people who are high in NCC but low in NC are less likely to reject conspiracy theories about COVID-19 than high NCC & high NC people, low NCC & high NC people, and low NCC & low NC people*).

To further test whether there were different types of respondents as proposed in Table 1, we performed a principal component analysis. This cluster analysis did not accurately summarize the data to support the notion of distinct types of respondents based on NC, NCC, COVID-19 Knowledge, Conspiracy Rejection, and media use (see Supplementary Fig. 1 in Appendix B). Therefore, the current results also did not support **hypothesis 1c** (i.e., *people who have relatively low NC rely more on new media and informal sources to acquire new information about COVID-19*).

**Figure 1:**
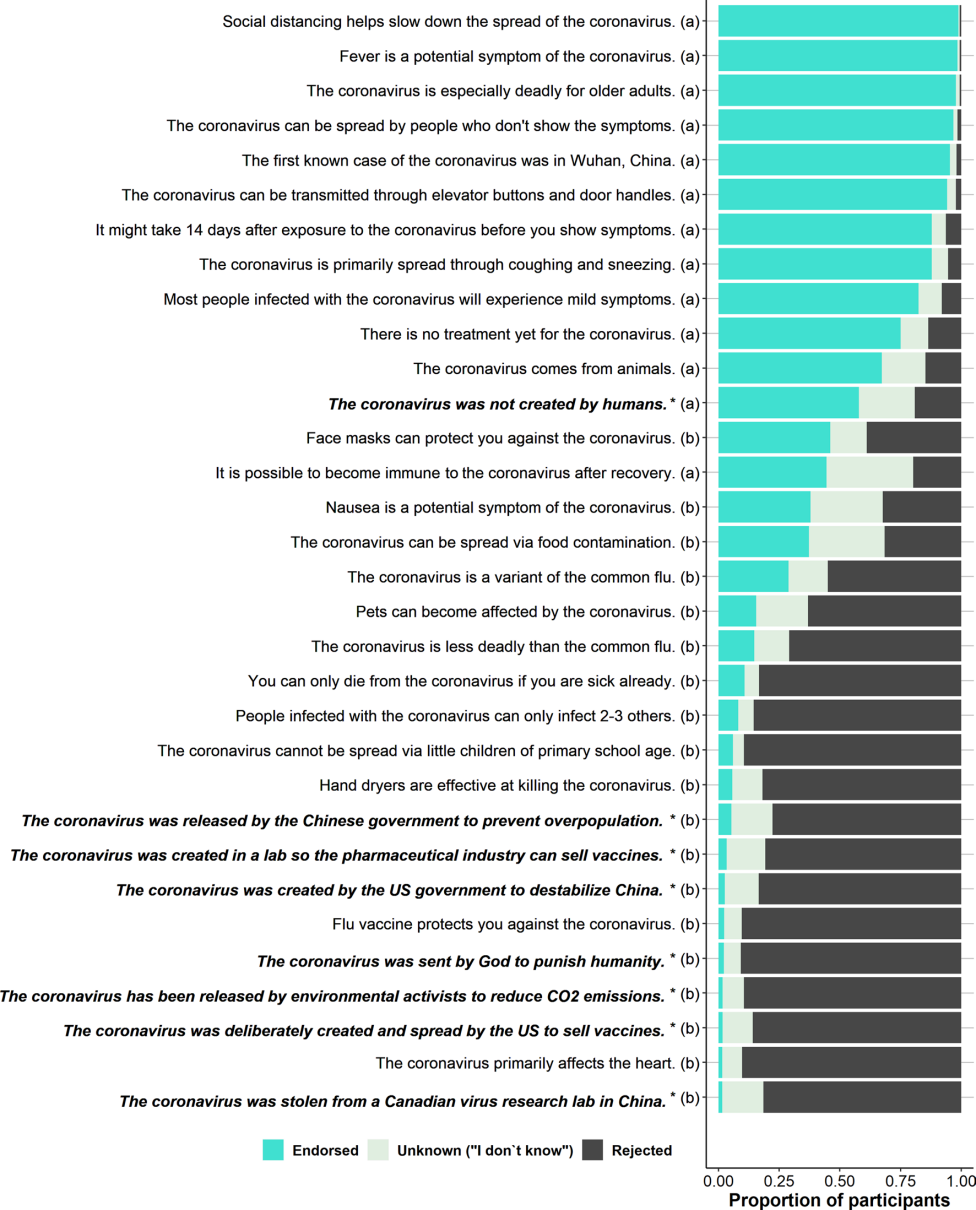
COVID-19 knowledge test**.** Proportions of respondents (N = 501) per answer option per item of the COVID-19 Knowledge Test**.** (**a**) = true statement, (**b**) = false statement, *conspiracy statement. See Appendix C for comparisons between groups based on NC and NCC scores **Cognitive motivation.** To compare the results of the COVID-19 Knowledge Test between different NC and NCC combinations we extracted four subsets from our sample. We used a median split to determine whether a respondent was high or low in NC. Whether respondents were high or low in NCC was based on the top and bottom quartiles of the NCC scores, a procedure suggested by the authors of the NCC test^16^. The discrepancy in categorization methods of NC and NCC groups (i.e., median split and outer quartiles) was chosen for pragmatic reasons. That is, excluding the data of two independent measures’ interquartile ranges would vastly diminish the number of observations per cell. Group sizes were 61 (Hi-NC, Hi-NCC), 78 (Hi-NC,Lo-NCC), 61 (Lo-NC, Hi-NCC), and 52 (Lo-NC, Lo-NCC). An important consideration here is that these groups were solely categorized for descriptive purposes and were not used in the inferential analyses. The descriptive results of the NC and NCC scales and subscales can be found in Supplementary Table 6 in Appendix A.

### Descriptive statistics

#### COVID-19 knowledge

Respondents’ knowledge of COVID-19 was approximated based on their personal evaluation of 24 correct and incorrect COVID-19-related statements (see Fig. 1; internal-consistency factor: ωh = 0.71). The highest consensus amongst respondents was yielded by the statement “*Social distancing helps slow down the spread of the coronavirus*” that was endorsed by about 99% of the respondents (three respondents rejected this claim and three respondents indicated not to know whether this statement was true or not). The statement that yielded the highest level of uncertainty (36.0%) was *“It is possible to become immune to the coronavirus after recovery.”* On average, respondents were better at judging true statements to be true (83.3%) than at rejecting false statements (66.7%; see Table 3). Respondents were also more likely to respond with “I don’t know” when evaluating false statements compared to true statements, showing more ignorance. These results indicate that the average level of COVID-19 knowledge in our sample was fairly high in this early phase of the pandemic.

**Table 3:** Descriptive statistics COVID-19 knowledge test (N = 501).

Item veracity	Knowledge test answers	Mean percentage	SD	95% CI	Min	Max
True statements (12 items)	*"I am sure this is true"*	56.1	22.0	[54.2, 58.1]	0	12/12 items
	*"I think this is true"*	29.4	19.0	[27.7, 31.1]	0	12/12 items
	*"I don't know"*	8.2	10.3	[7.3, 9.1]	0	10/12 items
	*"I think this is not true"*	4.4	6.1	[3.9, 4.9]	0	4/12 items
	*"I am sure is not true"*	1.9	4.1	[1.5, 2.2]	0	3/12 items
False statements (12 items)	*"I am sure this is true"*	4.5	7.2	[3.8, 5.1]	0	5/12 items
	*"I think this is true"*	13.5	10.7	[12.5, 14.4]	0	7/12 items
	*"I don't know"*	14.4	13.4	[13.2, 15.5]	0	11/12 items
	*"I think this is not true"*	25.0	16.2	[23.5, 26.4]	0	10/12 items
	*"I am sure is not true"*	42.7	20.5	[40.9, 44.5]	0	11/12 items
Conspiracy statements (7 items)	*"I am sure this is true"*	0.3	2.5	[0.1, 0.5]	0	2/7 items
	*"I think this is true"*	2.4	7.9	[1.7, 3.1]	0	5/7 items
	*"I don't know"*	13.1	24.7	[11.0, 15.3]	0	7/7 items
	*"I think this is not true"*	20.5	28.1	[18.1, 23.0]	0	7/7 items
	*"I am sure is not true"*	63.6	37.7	[60.3, 66.9]	0	7/7 items
Negated conspiracy statement (1 item)	*"I am sure this is true"*	28.7	45.3	[24.8, 32.7]	Not applicable for a single item	
	*"I think this is true"*	29.1	45.5	[25.2, 33.1]		
	*"I don't know"*	23.0	42.1	[19.3, 26.6]		
	*"I think this is not true"*	11.8	32.3	[9.0, 14.6]		
	*"I am sure is not true"*	7.4	26.2	[5.1, 9.7]		

#### COVID-19 conspiracy evaluation

We examined respondents’ evaluation of conspiracy theories by presenting eight COVID-19-related statements containing nefarious motives by groups of powerful agents. Respondents showed high rejection of the eight conspiracy statements (internal-consistency factor: ωh = 0.89): 373 respondents (74.5%) categorically rejected all conspiracy statements, 91 respondents (18.2%) endorsed one conspiracy statement, 21 respondents (4.2%) endorsed two conspiracy statements, and only 16 respondents (3.2%) endorsed three or more conspiracy statements. Interestingly, the level of uncertainty was much higher than the level of endorsement: 78 respondents (15.6%) were unsure about the veracity of one conspiracy statement and 120 respondents (24.0%) were unsure about two or more conspiracy statements. However, the statement that explicitly denied human motives, *“The coronavirus was not created by humans,*” was rejected by 96 respondents (19.2%) and 115 respondents did not know the answer (23.0%). This means that 19.2% of our respondents thought the coronavirus was created by humans. In comparison, each more specific statement implying human motives was endorsed on average by 14 respondents (2.7%) and 65 respondents (13.1%) did not know the answer. Table 3 shows the descriptive statistics of the COVID-19 Knowledge Test.

Supplementary Tables 8–11 (in Appendix A) present comparative statistics of the COVID-19 Knowledge Test between the four NC and NCC groups. The most striking contrasts can be observed between respondents high and low in NCC, showing systematic differences in the use of *“I am sure*”, *“I think”*, and *“I don’t know”* answers, for the knowledge statements as well as the conspiracy statements. Particularly, respondents high in NCC more often respond with “*I am sure*”, whereas respondents low in NCC tend to respond with *“I think*” and *“I don’t know”* relatively more often. For example, low NC/high NCC-respondents evaluated an average of 64.2% (95% CI[59.9, 68.5] of true statements with “*I am sure this is true*”, whereas low NC/low NCC-respondents evaluated an average of 47.8% (95% CI[40.5, 55.0]) of true statements with *“I am sure this is true*” (see Supplementary Table 7 in Appendix A). In contrast, low NC/high NCC-respondents evaluated 23.2% (95% CI[19.0, 27.4] of true statements with “*I think this is true*”, whereas low NC/low NCC-respondents evaluated 32.5% (95% CI[26.7, 38.3]) of true statements with *“I think this is true”*. Additionally, low NC/low NCC-respondents more often used the *“I don’t know”* response: 12.5% (95% CI[8.5, 16.5]) compared to 4.6% (95% CI[3.2, 6.1]) by the low NC, high NCC group. Similar trends are evident in the false statement category in Supplementary Table 8 in Appendix A.

Conspiracy endorsement across the four NC and NCC types was low (i.e., close to 0%, see Supplementary Table 9 in Appendix A). That said, there was a large difference in the use of the answer “*I am sure this is not true”* with regard to conspiracy statements between high NC/high NCC-respondents and low NC/low NCC-respondents: 80.6% (95% CI[72.7, 88.5]) chance and 50.8% (95% CI [40.2, 61.5]) chance, respectively. Moreover, low NC/low NCC-respondents also showed a greater tendency to answer “*I don’t know”* with 17.9% (95% CI[10.1, 25.6]) as compared to high NC/high NCC-respondents with 4.4% (95% CI[-0.2, 9.1]) chance to answer *“I don’t know*”. This suggests respondents low in NC and low in NCC were less likely to make explicit truth claims, and therefore, less likely to reject conspiracy statements.

#### Media use related to COVID-19: use of sources

**Figure 2:**
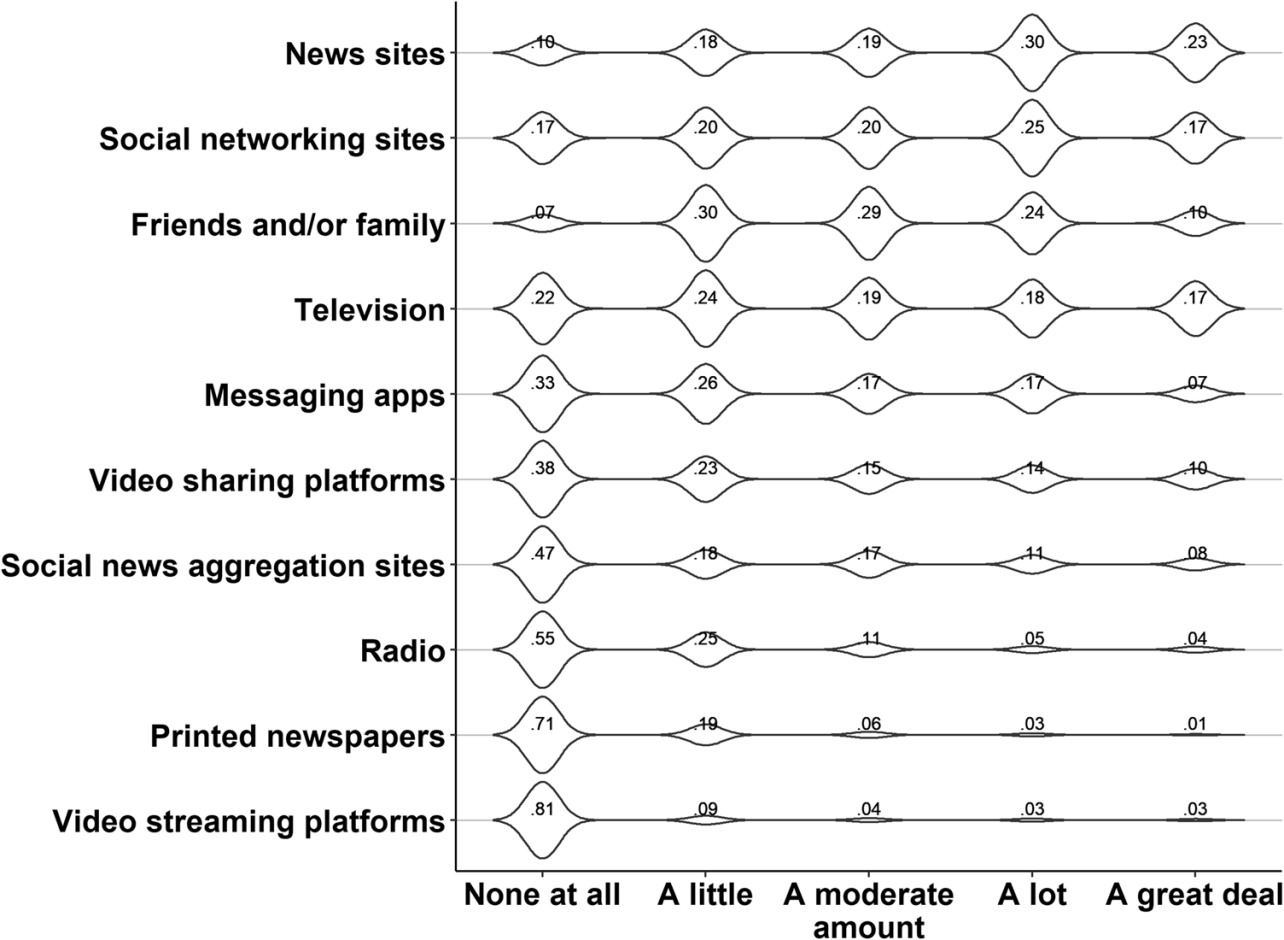
Media use wave 1**.** Proportion of respondents (N = 501) that made use of a particular source to acquire COVID-19 information.

News sites, social networking sites, and television broadcasts are the primary sources of COVID-19 information in our sample (see Fig. 2). Traditional media, such as printed newspapers and radio broadcasts, are scarcely utilized. Almost all respondents (93%) indicated to rely on friends and family as sources of information. Yet this source type was not consulted frequently. Only 34% of the respondents indicated to rely on friends and family ‘a lot’ or ‘to a great deal’. This suggests that friends and family were more important as secondary rather than primary source of COVID-19 information, despite being the most used source.

#### Search for information

Many respondents (73.8%) actively searched for updates on COVID-19 (see Table 4). Interestingly, a large majority of respondents also coincidentally encountered COVID-19 news (79.4%) and was updated on COVID-19 by friends, family, or co-workers (78.4%). This implies that active and passive consumption of information are not mutually exclusive. Moreover, it suggests that not all COVID-19 news was necessarily desired. This is supported by the finding that 37.6% of the respondents ignored COVID-19 news at least occasionally.

**Table 4:** Conditions of COVID-19 news encounters.

Item	Strongly disagree	Somewhat disagree	Neither agree nor disagree	Somewhat agree	Strongly agree
I actively search for updates about the coronavirus	9.2	10.4	6.6	40.5	33.3
I coincidentally read news about the coronavirus when I`m browsing online	4.4	6.0	10.2	51.1	28.3
I get updates about the coronavirus from other people (friends, family, coworkers)	3.0	8.0	10.6	48.9	29.5
I ignore news about the coronavirus	62.5	24.8	7.6	4.6	0.6

Values represent percentages. N = 501.

**Figure 3:**
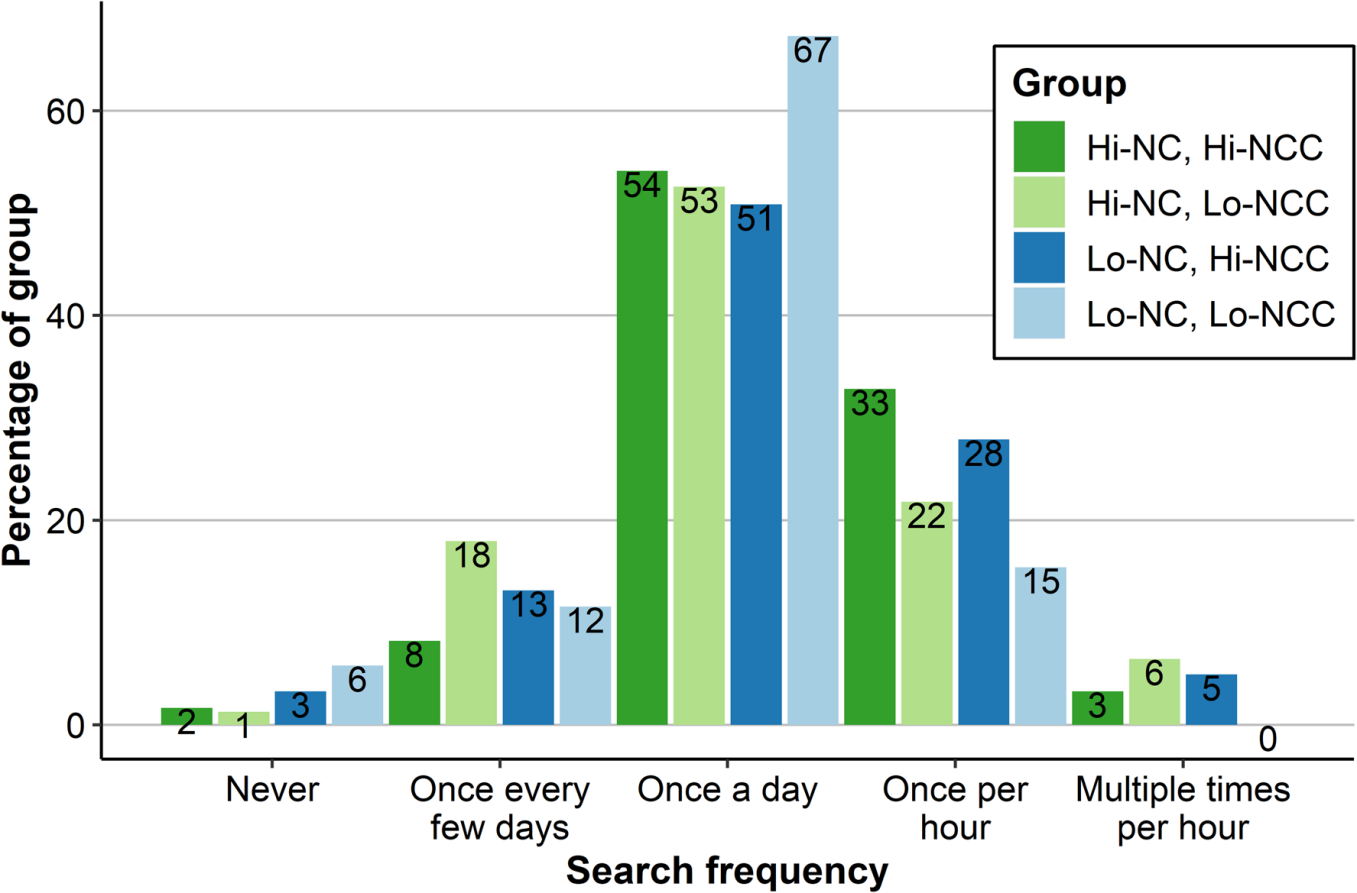
Media use frequency. COVID-19 Information Search Frequency Compared Across NC and NCC Types.

Most respondents actively searched for updates on COVID-19 once a day (53.7%), followed by once per hour (23.4%), once every few days (14.8%), multiple times per hour (5.4%), and never (2.8%). Thus, a large majority of respondents (82.5%) was engaged in COVID-19 information seeking behaviour on a daily basis or more frequently. Search frequencies seem to be relatively similar across NC and NCC groups (see Fig. 3).

Nearly all respondents (96%) actively searched for COVID-19 related information due to a desire to be informed, whereas half of the respondents (51.7%) also expressed a desire to educate others about COVID-19 (see Table 5). Searching behaviour was more driven by concerns about the health of close friends or relatives (92.4%), than by concerns regarding the health of the general public (84.4%) or respondents’ own health (66.8%).

**Table 5:** Motivations for COVID-19-related information acquisition.

Item	Strongly disagree	Somewhat disagree	Neither agree nor disagree	Somewhat agree	Strongly agree
I want to be informed about the coronavirus	1.2	1.2	1.6	30.1	65.9
I want to educate others	7.0	14.2	27.1	28.3	23.4
I worry about my own health	5.2	16.0	12.0	38.5	28.3
I worry about the health of people close to me	1.6	3.4	2.6	26.9	65.5
I worry about the health of the general public	3.0	4.0	8.2	39.9	44.9

Values represent percentages. N = 501.

## Discussion

### Acquisition of information about COVID-19

We tested preregistered hypotheses about the cognitive antecedents of COVID-19 information acquisition and processing. Specifically, we hypothesized that respondents would engage in certain information seeking behaviour based on their NC and NCC scores. Contrary to our hypothesis, cognitive motivation, as measured by NC and NCC, was not related to the frequency or type of media use related to COVID-19 information (i.e., hypotheses 1b and 1c).

Descriptive analyses showed that a large majority actively searched for COVID-19 related news while also encountering this news accidentally. The frequency of searching for COVID-19 news updates was daily for the majority of information seekers. Also interesting was that some respondents actively ignored information about the virus, even early in the pandemic.

We also investigated the underlying circumstances and motivations for COVID-19 information acquisition and general media use. The main motivations to acquire new information were the personal desire to be informed and, to a lesser extent, to be able to educate others. Moreover, health concerns related to the risks of COVID-19 were mainly directed at close friends or relatives, and more altruistically, the general public’s health instead of personal health. It is important to recall once more that this information seeking behaviour was measured on March 27, in a relatively early phase of the pandemic in the countries from which our sample originates.

### Interpretation of information

As for the acquisition of COVID-19 related information, we also expected NC and NCC to influence the interpretation of information. More specifically, we hypothesized that people high in NC have more factual knowledge about COVID-19 than people low in NC (i.e., hypothesis 1a), people high in NCC feel more certain about their knowledge than people low in NCC (i.e., hypothesis 2), and we expected weaker rejection of conspiracy theories in people low in NC and high in NCC (i.e., hypothesis 3). Before testing these hypotheses, a confirmatory factor analysis indicated that the COVID-19 Knowledge Test can be used to measure factual knowledge on COVID-19 as well as belief in conspiracies about the virus.

#### COVID-19 knowledge and confidence

Congruent with hypothesis 1a, a regression analysis showed that NC was positively related to COVID-19 knowledge. People high in NC appeared to be more knowledgeable about the virus and the situation. NCC was also positively related to COVID-19 knowledge and we found an interaction effect of NC and NCC on COVID-19 knowledge. Participants who were both high in NC and NCC showed higher levels of COVID-19 knowledge (i.e., accurate answers with higher confidence). Descriptive analyses suggest that, in line with hypothesis 2, people high in NCC are more certain about their responses to knowledge statements than people low in NCC. This pattern was found for true as well as false statements.

The descriptive statistics showed a clear distinction in the processing of true versus false statements in the COVID-19 Knowledge Test. Respondents showed higher accuracy in their evaluations of true statements. These results suggest COVID-19 information seekers tend to be less eager to make an explicit judgement about the veracity of false information. This may be rooted in the idea that false statements are more likely to be unrecognized or unknown. From an intellectually honest standpoint, it is fair to say “I do not know whether this is true.” Interestingly, this introduces an epistemological bias: false information is less likely to be refuted than correct information is to be endorsed.

#### COVID-19 conspiracy rejection

We hypothesized that conspiracy beliefs could be predicted by an interaction of low NC and high NCC (i.e., Hypothesis 3). That is, a combination of high confidence in personal knowledge (i.e., high NCC) without a need for intellectual activities and acquisition of new information (i.e., low NC) would, hypothetically, render a person more likely to engage in conspiracy thinking. However, we did not find this interaction effect. It might have been difficult to uncover the true relationship between NC, NCC, and conspiracy beliefs due to the very low number of respondents endorsing the conspiracy items in our sample (i.e., 4.8% average chance to endorse conspiracy statements; 2.7% chance after exclusion of item “The coronavirus was (not) created by humans.”). The ability to successfully reject conspiracy theories (with high confidence) was associated with a high level of COVID-19 knowledge as evidenced by a positive correlation between COVID-19 knowledge and rejection of conspiracy theories, which is consistent with the findings from previous studies^18^.

An alternative way to assess support for conspiracy thinking is to assess how many respondents endorsed at least one of the conspiracy statements. On this measure, 91 respondents (18.2%) endorsed one of eight conspiracy statements and 37 respondents (7.4%) endorsed two or more conspiracy statements. Another aspect of the conspiracy evaluations that grasped our interest was the relatively high level of uncertainty: 78 respondents (15.6%) were unsure (i.e., they answered ‘I don’t know’ when asked to indicate whether the items were true) about one conspiracy statement and 120 respondents (24.0%) were unsure about two or more conspiracy statements. Thus, a large percentage of respondents (about 40%) were not able to make an explicit truth claim about at least one of the conspiracy statements (i.e., ‘I am sure this is true/not true’, ‘I think this is true/not true’). Perhaps, respondents who evaluate conspiracy statements with ‘I don’t know’ are more susceptible to adopt novel conspiracy beliefs, considering they do not reject conspiracy ideas at face value. Alternatively, respondents may feel more comfortable to withhold beliefs about novel ideas. Therefore, they are possibly more reluctant to make explicit judgements about surreal conspiracy claims, which tend to be more novel and yield less recognition.

Considering the timeline of the COVID-19 situation, we reckoned it was important to perform a follow-up survey. Three months after the first wave, we launched a second wave of the survey among the original sample. We addressed the following research questions: How do COVID-19 knowledge and endorsement of conspiracy theories change over time? What is the relation between these changes, NC, and NCC? How are NC and NCC associated with adherence to governmental measures regarding COVID-19? And finally, what are the most respected organizations and sources for obtaining information and guidelines about COVID-19 and how is this related to NC and NCC?

## Wave 2

### Method

#### Respondents

On July 1 of 2020, we sent out a Wave 2 survey to those individuals who had participated in Wave 1. Three-hundred-and-fifty-two respondents from Wave 1 participated in Wave 2. The data for ten respondents were removed from the analysis because they spent too much time on the COVID-19 Knowledge Test (z-score > 3.29) or failed the catch question. Sixteen respondents did not meet the inclusion criteria for Wave 1 and were therefore also excluded from the Wave 2 analyses. The final sample size was 326 (65% of the original sample, 154 females). Respondents’ ages ranged from 18 to 70 (*M* = 32.44, *SD* = 12.06). See Supplementary Tables 12–16 in Appendix A for descriptive statistics regarding the career fields, level of education, nationality, native language, and gender identity of our Prolific sample. On average, the survey took 15 min to complete. Respondents were paid £1.88, amounting to an average hourly fee of £7.50. All respondents were fluent in English according to the Prolific database.

#### Materials and procedure

Wave 2 comprised (1) an updated version of the COVID-19 Knowledge Test, (2) open-ended questions about the origin, symptoms, measures, and societal impact of COVID-19, (3) questions regarding the frequency of media use related to COVID-19 news, (4) questions regarding the importance of a range of sources for acquiring COVID-19 knowledge, and (5) adherence to government-imposed measures.

The COVID-19 Knowledge Test was updated. One item was moved from the false to the true statement category, because new scientific insight proved this statement to be true rather than false, and eight new items were added. As a result, the updated COVID-19 Knowledge Test consisted of 16 true statements and 16 false statements. We made no changes to the conspiracy statements. To assess media use, respondents indicated how frequently they made use of 18 (categories of) sources to acquire COVID-19 knowledge on a 7-point scale (1 = *I don’t use this source*, 7 = *multiple times per day*). The complete survey (https://osf.io/vrbpw/) as well as our preregistration (https://osf.io/mp2ua/) can be viewed online on OSF. All the measures (except the open-ended questions) from the second survey are displayed in Table 6. The data from the open-ended questions about the origin, symptoms, measures, and societal impact of COVID-19 are reported in a supplementary appendix.

**Table 6:** Overview and descriptions of the measures in wave 2.

Category	Measure	Items	Description
Information processing	COVID-19 Knowledge	32	*Knowledge about COVID-19* in terms of accuracy and confidence
	Conspiracy Rejection	8	*COVID-19-related conspiracy rejections* in terms of accuracy and confidence
Media use	Frequency per source	18	The extent to which different types of sources are used to acquire information about COVID-19 (e.g., news sites, social networking sites, friends/family)
Behaviour	Adherence to measures	1	The degree of adherence to government-imposed measures against COVID-19
	Perceived importance of sources	8	The perceived importance of different sources and organizations with respect to acquiring new information and guidelines

The measures of Wave 2 were complementary to the Wave 1 measures. Again, we measured COVID-19 information processing and interpretation (COVID-19 Knowledge and Conspiracy Rejection), which allowed us to investigate temporal changes between Wave 1 and Wave 2. In addition, we added a more comprehensive media question to assess usage frequency per distinct media source. We also added two behaviourally focused questions about adherence to government-imposed measures and the perceived importance of different organizations that publish health regulations or guidelines to counter COVID-19. We used measures on cognitive motivation and demographic traits from Wave 1, because our Wave 2 sample 1 (N = 326) comprised a subset of our Wave 1 participants (N = 501).

## Results

Similar to the Wave 1 results, we will start with the report of our inferential analyses followed by descriptive analyses.

### Wave 1 and Wave 2 overall comparison

Did knowledge of COVID-19 and belief in conspiracy theories change between March and July 2020? To determine whether we could accurately quantify change in COVID19-Knowledge and Conspiracy Rejection factors, we used the longitudinal measurement invariance of the COVID19-Knowledge Test using data from all 326 individuals that participated in both waves. Because all items are categorical, however, not all default measurement invariance analysis steps could be undertaken. A model for configural invariance for example, would have to estimate 62 factor loadings, 248 thresholds, 62 residual variances, 2 latent factor variances, 2 latent factor means, and a covariance between the latent factors. If we allowed for correlated residual across measurement occasion, we would also estimate 62 such correlations. This number of parameters is too high to obtain stable estimates given our sample size. We therefore chose to fit an invariance 2-wave model of the COVID-19 Knowledge Test in which all factor loadings and thresholds were constrained to be equal, and in which residuals were freely estimated and allowed to correlate over time. All analyses were run using the R-package lavaan (Rosseel, 2012). In addition, all models were fitted using the diagonally weighted least squares (DWLS) estimator, robust standard errors, and a mean- and variance-adjusted chi-square test. The invariance 2-wave model showed sufficient fit to the data (χ^2^ (1939) = 2978.44, *p* < 0.001; CFI = 0.925; RMSEA = 0.041) which allowed an investigation of change in COVID-19 Knowledge and Conspiracy Rejection between waves using Latent Difference Scores (McArdle, 2009). Note that we did not pre-register the use of latent difference scores. We proposed regressing the factor-scores obtained on the Wave 2 data on the factor scores from Wave 1. However, the latent difference scores gave us a more precise estimate of change in the factors and the variance in this change. As we used latent difference scores, the relation between NC and NCC on the one hand and change in COVID19-Knowledge and Conspiracy Rejection on the other was investigated by regressing the latent difference scores for these two factors on the total NC and NCC scores. This Latent Difference Score model is an equivalent model to the 2-wave model and therefore has identical fit (χ^2^(1939) = 2978.44, *p* < 0.001; CFI = 0.925; RMSEA = 0.041). The results showed that there was no significant mean increase in either COVID-Knowledge (− 0.029 (0.050), *p* = 0.559) or Conspiracy Rejection (− 0.011 (0.053), *p* = 0.833). There was significant variance in both latent change factors (Knowledge: s2(se) = 0.447 (0.070), χ^2^(1) = 54.48, *p* < 0.001; Conspiracy: s2(se) = 0.520 (0.058), χ^2^(1) = 83.02, *p* < 0.001).

### Wave 1 and Wave 2 comparison based on NC and NCC

In addition to overall changes in *COVID-19 Knowledge* and *Conspiracy Rejection* from Wave 1 to Wave 2, we asked whether any changes could be associated with NC and NCC. Adding NC, NCC, and their interaction as predictors for the latent change scores of *COVID19-Knowledge* and *Conspiracy Rejection* showed that NC and NCC were both positively related to change in *COVID19-Knowledge* (NC: b(se) = 0.196 (0.076), beta = 0.253, *p* = 0.010; NCC: b(se) = 0.283 (0.076), beta = 0.366, *p* < 0.001), while NCC was also positively related to change in *Conspiracy Rejection* (b(se) = 0.188 (0.071), beta = 0.246, *p* = 0.008). Need for Cognition was not significantly related to change in *Conspiracy Rejection* (b(se) = 0.122 (0.066), beta = 0.160, *p* = 0.063). There was no significant interaction effect on change in either *COVID19-Knowledge* (b(se) = -0.113 (0.062), beta = -0.198, *p* = 0.068) or *Conspiracy Rejection* (b(se) = − 0.061(0.054), beta = -0.108, *p* = 0.266). In summary, both NC and NCC were associated with an increase in COVID-19 Knowledge from Wave 1 to Wave 2. NCC was also a predictor of an increase in rejectance of conspiracy statements from Wave 1 to Wave 2.

### COVID-19 knowledge and compliance behaviour

The relation between COVID19-Knowledge and Conspiracy Rejection at Wave 2 and (1) adherence to government-imposed measures and (2) the perceived importance of each information source was investigated using a path model. In this model, the eight items on different news sources, and adherence to measures were endogenous or dependent variables, while the Wave 2 factor scores were exogenous or independent variables. *COVID-19 Knowledge* and *Conspiracy Rejection* were added to this model as predictors. Results show that this path model sufficiently fitted the data (χ^2^(694) = 1071.78, *p* < 0.001; CFI = 0.943; RMSEA = 0.045). In addition, we found that COVID19-Knowledge at Wave 2 was significantly positively related to adherence behaviour at Wave 2 (b(se) = 0.225 (0.065), beta = 0.271, *p* = 0.001). All other relations were non-significant.

To investigate the relationships of NC and NCC with adherence to government-imposed measures, the perceived importance of respected sources, participant age (as covariate factor) and media use, we computed an additional network model (see Supplementary Fig. 6 in Appendix B). The R package ‘bootnet’ and the complementary function ‘estimateNetwork()’ with the “EBICglasso” method (i.e., a Gaussian Markov random field estimation based on graphical LASSO and an extended Bayesian information criterion to select parameters) were used (Epskamp, Borsboom, & Fried, 2018). Both NC and NCC were not related to the perceived importance of respected sources, adherence to measures, or media use. However, NC and NCC were weakly negatively correlated and NCC was positively correlated to participant age.

### Descriptive analyses

#### COVID-19 knowledge

On average, 73.3% of the true statements were correctly endorsed, whereas 67.9% of the false statements were correctly rejected (see Supplementary Table 17 in Appendix A; internal-consistency factor: *ωh* = 0.80).The largest difference in responses was yielded by the item “*Face masks can protect you against the coronavirus.*” That is, this statement was endorsed by more respondents in Wave 2 (30.7%) than in Wave 1 (10.1%). This specific item also explains the overall increase of “*I am sure this is true*” judgements in the false statement category (see Table 7). The overall evaluations of the COVID-19 Knowledge Test statements for Wave 2 are presented in Supplementary Fig. 2 in Appendix B, and the latent difference scores between Wave 1 and Wave 2 per item are presented in Supplementary Fig. 3 in Appendix B.

**Table 7:** Comparison of COVID-19 knowledge test answers between wave 1 and wave 2 (Within-group and Identical Items Only; N = 326).

Item veracity	Knowledge test answers	Wave 1		Wave 2		Diff
		Mean percentage	95% CI	Mean percentage	95% CI	
True statements (12 items)*	*"I am sure this is true"*	57.0	[54.6, 59.4]	53.2	[50.6, 55.9]	− 3.8
	*"I think this is true"*	28.6	[26.5, 30.6]	31.2	[28.9, 33.5]	+ 2.6
	*"I don't know"*	8.4	[7.3, 9.5]	8.6	[7.5, 9.6]	+ 0.2
	*"I think this is not true"*	4.3	[3.6, 4.9]	5.1	[4.2, 5.9]	+ 0.8
	*"I am sure this is not true"*	1.7	[1.3, 2.2]	1.9	[1.1, 2.8]	+ 0.2
False statements (11 items)*	*"I am sure this is true"*	4.7	[3.9, 5.6]	6.6	[5.7, 7.5]	+ 1.9
	*"I think this is true"*	13.4	[12.2, 14.7]	14.9	[13.8, 16.1]	+ 1.5
	*"I don't know"*	13.9	[12.5, 15.4]	13.5	[12.0, 15.0]	− 0.4
	*"I think this is not true"*	23.8	[22.0, 25.6]	22.1	[20.2, 23.9]	− 1.7
	*"I am sure this is not true"*	44.1	[41.7, 46.5]	42.9	[40.5, 45.3]	− 1.2
Conspiracy statements (7 items)	*"I am sure this is true"*	0.3	[0.0, 0.6]	0.9	[0.4, 1.4]	+ 0.6
	*"I think this is true"*	2.5	[1.6, 3.3]	3.2	[2.1, 4.4]	+ 0.8
	*"I don't know"*	14.2	[11.4, 17.0]	11.3	[8.8, 13.7]	− 2.9
	*"I think this is not true"*	19.6	[16.6, 22.6]	19.4	[16.5, 22.3]	− 0.2
	*"I am sure this is not true"*	63.5	[59.3, 67.6]	65.2	[61.1, 69.2]	+ 1.7
Negated conspiracy statement (1 item)	*"I am sure this is true"*	28.5	[23.6, 33.4]	26.7	[21.9, 31.5]	− 1.8
	*"I think this is true"*	27.9	[23.0, 32.8]	26.7	[21.9, 31.5]	− 1.2
	*"I don't know"*	23.0	[18.4, 27.6]	22.7	[18.1, 27.3]	− 0.3
	*"I think this is not true"*	13.2	[9.5, 16.9]	12.9	[9.2, 16.5]	− 0.3
	*"I am sure this is not true"*	7.4	[4.5, 10.2]	11.0	[7.6, 14.5]	+ 3.7

This table shows comparative descriptives of the COVID-19 Knowledge Test of Wave 1 (March 27, 2020) and Wave 2 (July 1, 2020). The percentages are based on a subset of participants that participated in both surveys. The items that were not presented to participants in Wave 1 were removed. One item of the false items was moved to the correct statement category in Wave 2, and therefore also removed from this comparison. Differences in percentages that show overlap between CIs are presented in grey font colour.

#### COVID-19 conspiracy evaluation

More than half of the respondents (57.4%) either rejected all eight conspiracy theories or gave neutral answers both at Wave 1 and Wave 2 (internal-consistency factor Wave 2: *ωh* = 0.89). A far smaller group of 46 respondents (14.1%) accepted at least one conspiracy theory both at Wave 1 and Wave 2. This means that belief in conspiracy theories changed between Wave 1 and Wave 2 for 28.5% of the respondents. This change contained either a shift from rejection of all conspiracy theories at Wave 1 to endorsement of at least one conspiracy item at Wave 2 (16.3%), or the other way around (12.3%). Moreover, 39.3% of respondents could not explicitly reject all of the conspiracy statements both at Wave 1 and Wave 2, whereas 35.2% did reject all conspiracy statements at Wave 1 and Wave 2. The remaining group showed changes on this measure: 12.6% of respondents could not reject all conspiracy statements at Wave 1, and 12.9% could not reject all conspiracy statements at Wave 2.

#### Media use

Supplementary Fig. 4 in Appendix B shows the frequencies with which different sources are consulted for COVID-19 information. Evidentially, the nature and frequency of COVID-19 information acquisition is highly variable across respondents in our sample. That said, the source ‘friends, relatives, and co-workers’ yielded the highest proportion of respondents who reported to use it ‘multiple times per day’. This suggests that colloquial conversations play a prominent role in the exchange of COVID-19 information. On a daily basis, the most widely used sources of COVID-19 information are news broadcasts (e.g., “BBC”, “CNN”), television/radio, and newspapers (incl. digital). Furthermore, Google and real-time statics sites are also frequently used to obtain or encounter COVID-19 news. Within the category of social media, Facebook and Twitter are the most popular sources of COVID-19 news. Overall, respondents in our sample mainly rely on conventional media to obtain information about COVID-19. However, they also encounter information colloquially and when they use social media.

#### Perceived importance of sources

Supplementary Fig. 5 in Appendix B presents the perceived importance of different sources and organizations with respect to acquiring COVID-19 information and guidelines. The majority of respondents reported the World Health Organization (WHO) to be either important or very important (69%), whereas 10% of respondents perceived the WHO as unimportant. Furthermore, national health organizations were also reported as important or very important by the majority of respondents (78%), whereas only 4% perceive their national health organization as unimportant. After health organizations, governments (58%), heads of governments (51%), general practitioners (51%), (former) COVID-19 patients (47%), and relatives/family (40%) are perceived as important or very important by approximately half of the respondents. Moreover, leaders of respondents’ chosen political parties are perceived as unimportant by 40%, whereas only 26% perceive their party leaders as important or very important. Altogether, the WHO and national health organizations are highly valued in our sample, whereas political party leaders are perceived as less important.

#### Adherence behaviour

We also investigated the respondents’ adherence to government-imposed measures. Most respondents (48.5%) reported to ‘always’ follow the measures. The second largest group (37.1%) reported to ‘often’ follow the measures. A smaller group (9.8%) reported to ‘sometimes’ follow the news. The smallest group reported to either ‘rarely’ (2.8%) or to never (1.8%) follow the measures. Only four respondents (1.2%) appeared to be noncompliant for other reasons than inadequacy of their government. Overall, the provided explanations indicated that low adherence to measures can mainly be attributed to a lack of governmental guidance or actions. See the Supplementary Examples and responses to the open-ended question about adherence in Appendix D.

## Discussion

The second wave enabled us to address potential temporal-induced changes in the evaluation of the COVID-19 Knowledge Test statements. We found no considerable changes in COVID-19 knowledge and conspiracy beliefs from March 27 to July 1. However, one of the statements was a remarkable exception: *“Face masks can protect you against the coronavirus.”* respondents judged this statement to be true more often in the second wave. Possibly, this change in opinions reflects a behavioural shift on a societal level. That is, while governments have endorsed or imposed the use of face masks to mitigate the spread of the virus, citizens may have adopted a more positive sentiment towards the use of face masks. Perhaps, citizens tend to believe that face masks are used for self-protection rather than for altruistic purposes. In addition, governmental ads and guideline updates on wearing face masks may have increased explicit recognition of this statement, which likely has induced a more positive affect. Thus, increasing the likelihood that respondents evaluate this statement to be true in Wave 2. Indeed, previous studies have shown that explicit recognition increases positive affect towards priorly exposed stimuli (Brooks & Watkins, 1989; Fang, Singh, & Ahluwalia, 2007; Newell & Shanks, 2007; Stafford & Grimes, 2012; Wang & Chang, 2004).

Despite overall factual knowledge about COVID-19 remaining relatively unaffected, both NC and NCC (assessed in Wave 1) were positively associated with latent difference scores on the items between Wave 1 and Wave 2. This suggests that respondents high in NC or NCC were more likely to increase their knowledge about COVID-19 from Wave 1 to Wave 2. Considering that respondents high in NC are more likely to engage in information-acquiring activities, it is not surprising that they were more likely to change their views over time. However, the positive relation we found between the difference scores and NCC appears less intuitive. Theoretically speaking, high NCC individuals would be more likely to remain unswayed by new information and be more prone to hold on to initial beliefs. As discussed in the introduction, NCC consists of two processes, *seizing* and *freezing*. As long as people are in the seizing phase, they will continue to collect information and compare it to the interpretation they already have to achieve closure. Once they are confident in their interpretation, they are in the freezing phase and the interpretation process will be terminated. It is quite possible, given the volatile nature of the pandemic, that many of our respondents had not yet reached the freezing phase and were still comparing their initial interpretation to alternatives. We note, however, that without explicit measures of seizing and freezing, the hypothesis becomes unfalsifiable.

Another potential explanation for the positive relation of NCC on our measure of *Conspiracy Rejection* over time might be rooted in the high confidence level of high NCC participants. That is, if both high and low NCC groups would gain news insight between Wave 1 and Wave 2 about the origin and motives related to COVID-19, high NCC participants would be relatively more likely to answer with “I am sure this is not true” at Wave 2, whereas low NCC participants would be relatively more likely to answer with “I think this is not true” at Wave 2. Therefore, the latent difference scores for high NCC participants would be higher (i.e., + 2) as compared to low NCC participants (i.e., + 1.) In addition, doubts about the reprehensibility of the conspiracy statements at Wave 1 may have been mitigated at Wave 2, because participants were debriefed about the reprehensibility of some of the statements (without mentioning which particular statements were false) after the first survey. Thus, during the first survey participants were probably more oblivious to the presence of the implemented false conspiracy statements and more prone to answer neutrally, whereas in the second survey participants were more cognizant of the potential presence of these false statements.

Compared to Wave 1, this second wave included three additional measures: (1) a more in-depth inquiry on the media use with respect to COVID-19, (2) an indication of perceived importance of different sources, and (3) an indication of adherence to government-imposed measures against COVID-19. We did not find relations of these variables with either NC or NCC.

## General discussion

We were interested in how cognitive motivation impacts the acquisition, processing, interpretation of information in a situation of great uncertainty, namely during early phases of the COVID-19 pandemic. In addition, we were interested in how cognitive motivation is associated with the updating of mental representations after a three-month interval. We will discuss our main findings regarding the relations, or absence thereof, we observed between our measures of cognitive motivation, NC and NCC, and COVID-19 knowledge, conspiracy rejection, media use, and behavioural measures. We will also highlight several additional noteworthy findings.

### Cognitive motivation

People differ with respect to the amount of pleasure they derive from their own thought processes and with respect to how motivated they are to quickly arrive at an interpretation of a state of affairs. The former is called the Need for Cognition, the latter the Need for Cognitive Closure^3,4,9,19^. We reasoned that people would engage in different behaviours regarding the gathering, processing, and interpretation of information based on their need for cognition and need for cognitive closure. We thought of NC and NCC as largely unrelated factors. Consistent with this, we found that NC and NCC exhibit a slightly negative correlation. This finding is in line with previous research^5^.

### COVID-19 knowledge

In line with our first hypothesis (see **H1a**), the regression analysis of Wave 1 and Wave 2 data showed a relationship between NC and COVID-19 Knowledge, in which higher NC predicts increased knowledge. Unexpectedly however, NCC was also associated with increased COVID-19 Knowledge. Therefore, people who are high in NC and/or NCC have more COVID-19 Knowledge (based on our measure of COVID-19). Confirmatory analysis of the differences between Wave 1 and Wave 2 scores also showed positive effects of both NC and NCC on COVID-19 Knowledge over time. People high in NC as well as people high in NCC showed an increase of knowledge (i.e., an increase of confidence and/or accurate knowledge). Thus, as might be expected, people who enjoy cognitive activities more (i.e., people high in NC), are more likely to increase their knowledge, as are people who are looking for a coherent interpretation of a state of affairs (i.e., people high in NCC).

The statement “It is possible to become immune to the coronavirus after recovery” was regarded as a true statement in the COVID-19 Knowledge Test. We are aware that this notion has been quite controversial during the beginning of the pandemic. Therefore, we provide additional rational for classifying the statement as true. At the time of the survey there was no scientific consensus on whether people could develop long-term immunity to the SARS-CoV-2 virus (i.e., the cause of COVID-19). For example, there were known cases of recovered patients who remained viral positive or even relapsed^20^. However, research also showed that that a primary SARS-CoV-2 infection in rhesus macaques could protect from reinfection, and there was evidence of SARS-CoV-2 specific antibodies in humans as well^21,22^. Evidently, there was *a possibility* to become immune after recovery (at least temporarily). Moreover, governments such as in the UK and the Netherlands mentioned ‘herd immunity’ as a natural by-product of the pandemic (which was a controversial sentiment because achieving herd immunity without vaccinations would, contrary to the goal, cost many lives)^23^. Thus, there was both evidence and (in some countries) governmental acknowledgment of the possibility of immunity.

### Conspiracy theories

We hypothesized that people low in NC but high in NCC are more likely to engage in conspiracy beliefs (see **H3**). However, we did not find this interaction effect. Instead, both NC and NCC are positively related to the rejection of conspiracy theories. NCC also proved to be associated with an increase in rejection of conspiracy theories over time, from Wave 1 to Wave 2. The ability to successfully reject conspiracy theories is associated with more knowledge about COVID-19 as well. The average chance of endorsing a conspiracy theory was about 2.7% at Wave 1 and Wave 2 (excluding the item “The coronavirus was not created by humans”). Thus, the endorsement of conspiracy theories is marginal in our sample. Alternatively, we found that about a quarter of participants endorsed at least one of eight conspiracy theories. However, we have to consider this would also inflate the potential influence of response biases such as the acquiescence bias (i.e., ‘yea-saying’) with a factor of eight (i.e., the number of conspiracy items). Obviously, not all conspiracy theories are wrong. Watergate, for example, was an actual conspiracy. In this sense, it is perhaps not surprising that a sizable portion of respondents were unable to refute all conspiracy statements. That said, considering results of both Wave 1 and Wave 2, only 14.1% of participants endorsed at least one conspiracy theory at both waves, which is still considerably lower than conspiracy endorsement found in other studies^24^. For example, Oliver and Wood (2014) found that 55% of their participants endorsed at least one conspiracy theory, in a study by Gombin (2013) this was 50.3%^25,26^. However, we have to be careful making direct comparisons considering the differences in specific ideologies and content conveyed by conspiracy statements between studies, as well as differences in the demographic traits (e.g., nationality, political affiliation) of the samples.

### Media use

We expected to observe different types of information seeking behaviours based on variations in NC and NCC. First, we expected NC to predict the frequency of information gathering activities. Specifically, people high in NC would seek out new information more frequently than people low in NC (see **H1b**). However, regression analyses of Wave 1 data did not show relations of NC with COVID-19-related information searching frequency. Moreover, we expected that NC would predict the type of sources used as well (see **H1c**). However, none of the usage frequencies of the sources listed in our Wave 2 survey were related to NC. This means that, in our sample, NC did not predict the type or quality of sources used to acquire COVID-19.

### Clusters

Our confirmatory cluster analyses of the Wave 1 data failed to support the notion of four different groups of people based on the two measures of cognitive motivation, and our measures of COVID-1- knowledge, conspiracy thinking, and media use, as hypothesized in Table 1. This will be further discussed below.

### Self-reported behavioural measures

In our Wave 2 survey we added additional questions regarding the perceived importance of different sources and adherence to government-imposed measures, which did not show relationships with either NC or NCC. However, we did find that people with higher COVID-19 Knowledge (i.e., participants who performed better on the COVID-19 Knowledge Test) report more faithful adherence to measures.

### Limitations

The present study has several limitations. The outcome variable *COVID-19 Knowledge* can be seen as a combination of two conceptually independent constructs: the accuracy of the judgments (i.e., correct or incorrect) and the level of confidence in personal knowledge (i.e., “I am sure”, “I think”, “I don’t know”). From the participants perspective however, these constructs were not independent as they used a single ordinal five-point scale (i.e., 1 = “I am sure this is not true”, 2 = “ I think this is not true”, 3 = “I don’t know”, 4 = “I think this is true”, 5 = “ I am sure this is true”). We chose to use the original five-point scale in our inferential analyses and found main effects and interaction effects of NC and NCC on our measure of *COVID-19 Knowledge*. An important consideration here is that, based on our inferential analyses, we cannot conclude whether this effect is mainly caused by differences in judgement accuracy, judgement confidence, or both.

With that caveat, the comparative descriptive statistics of the COVID-19 Knowledge Test of Wave 1 data in Supplementary Tables 8–10 in Appendix A do suggest that NCC is a predictor of the level of confidence in people’s views about COVID-19. Respondents low in NCC showed lower levels of certainty compared to respondents who are high in NCC. More specifically, respondents who are low in NCC were relatively more likely to use the “I don’t know” and “I think” responses, whereas respondents high in NCC were relatively more likely to use the “I am sure” response. This suggests that high NCC individuals had more confidence in personal knowledge irrespective of the veracity of this knowledge. Either way, with the current design and analyses we cannot justify whether Hypothesis 2 (i.e., higher NCC predicts higher certainty) should be accepted or rejected. To properly analyse the relationship of NCC on confidence it is necessary to isolate the constructs of confidence and accuracy. Future research could introduce an experimental design that would allow for an appropriate separation of the two constructs.

Our cluster analysis showed no distinct groups based on *COVID-19 Knowledge*, *Conspiracy Rejection*, media use, and motivations to acquire news. A possible explanation might be that the data was not suited for the current cluster analysis method. More specifically, we used k-means clustering algorithms to compute principal components, which is based on two assumptions: the data should be spherical, and the clusters should be roughly equal in size. Perhaps, the current data did not meet these criteria. For example, if confidence levels are (partially) independent from the level of accuracy of knowledge this may result in non-spherical data; that is, one group would show more extreme scores with incorrect high confidence (i.e., 1) and correct high confidence scores (i.e., 5), whereas another group may have shown more centred scores (i.e., 2,3,4). In addition, the four hypothesized groups may be unequally represented in our sample. It is likely that a potential group of conspiracy endorsers would be relatively small compared to conspiracy rejecters. Thus, k-means clusters would show overlap, because this method does not account for large differences in group sizes.

Moreover, our sample might not have been representative. Our respondents displayed much factual knowledge about COVID-19, were eager to gather information about the virus as well as to educate others about it (> 50% of the respondents in Wave 1), and showed little endorsement for conspiracy theories regarding COVID-19. One could argue that perhaps our sample’s scores for NC and NCC are not comparable to the scores found in other studies. However, the NC and NCC scores found in our study correspond with previous research on NC (see Supplementary Table 7 in Appendix A for comparisons of the means and standard deviations)^27–32^.

A final limitation that should be discussed here is that the measure of adherence to government-imposed measures depended on the activities of one’s government. While we were mainly interested in the range of compliance to governmental guidelines and its relationship with NC and NCC, we are aware that this measure is likely confounded by the different political environments of the respondents. As shown in Appendix D, respondents who reported to rarely or never follow government-imposed guidelines predominantly explained that their governments were either inactive or inadequate with respect to countering the coronavirus.

## Conclusion

As might be expected in a state of high epistemic uncertainty, nearly three-quarters of our respondents actively searched for information about COVID-19. A large majority of the respondents was engaged in COVID-19 information seeking behaviour on a daily basis or even more frequently than that. Their primary sources were news sites, social networking sites, and television broadcasts. Traditional media, such as printed newspapers and radio broadcasts, were scarcely utilized. We speculate that one reason is that the latter do not provide information with the immediacy that the digital media allow.

Both Need for Cognition and Need for Cognitive Closure are associated with increased knowledge of COVID-19 and the ability to reject conspiracy theories about COVID-19. Thus, the extent to which our respondents acquired knowledge about COVID-19 is related to the extent to which they enjoy being engaged in their own thought processes and to the extent to which they seek a coherent interpretation of the information they have gathered. These two variables had an additive effect on levels of COVID-19 knowledge. While descriptive analyses suggest these relations can predominantly be explained by differences in confidence levels, future research should focus on an appropriate separation of the constructs of judgement accuracy and judgement confidence. Even though this study is framed within the context of the COVID-19 pandemic, our findings can be extended beyond this specific context. We expect cognitive motivations to be useful in distinguishing how people derive and elaborate on information from similar media environments as the ones we investigated in the current study.

## Supplementary information

Supplementary information 1.

Supplementary information 2.

Supplementary information 3.

Supplementary information 4.

## Data Availability

The datasets generated during and/or analysed during the current study are available in the Open Science Framework repository, https://osf.io/38b65/.
