# Supplementary material for: Gathering, processing, and interpreting information about COVID-19
Source: Sci Rep. 2021 Mar 22;11:6569. doi: 10.1038/s41598-021-86088-3 (PMC7985139; doi:10.1038/s41598-021-86088-3)
Supplement: Supplementary file 1 — Supplementary information 1. [file 41598_2021_86088_MOESM1_ESM.pdf]

Gathering, Processing, and Interpreting Information About COVID-19

Arnout B. Boot, Anita Eerland, Joran Jongerling, Peter P.J.L. Verkoeijen, and Rolf A. Zwaan

Appendix A- Supplementary Tables

Tables 1-5: Demographic Details of Prolific Research Participants (Wave 1, N = 501)

Table 1.

Participant Demographics: Career Field

Category	n	%
Student	100	20.0
Information Technology	64	12.8
Unemployed	56	11.2
Health Science and Health Care	46	9.2
Marketing, Sales, and Service	51	10.2
Education and Training	34	6.8
Science, Technology, Engineering, and Mathematics	47	9.4
Arts, Audio/Video Technology, and Communications	29	5.8
Business Management and Administration	22	4.4
Finance	16	3.2
Transportation, Distribution, and Logistics	14	2.8
Manufacturing	12	2.4
Law, Public Safety, Corrections, and Security	12	2.4
Government and Public Administration	12	2.4
Hospitality and Tourism	10	2.0
Human Services	9	1.8
Agriculture, Food and Natural Resources	7	1.4
Other: Retired	6	1.2
Other: Self-employed	3	0.6
Architecture and Construction	4	0.8
Other: Military	2	0.4
Other*	15	3.0

Note. * Other career fields reported by individual participants: associate, aviation, blogger, building management, clergy, educational publishing, freelancer, insurance, internet analyst, risk analyst, sex worker, sports, telecoms, and writing.

Table 2.

Participant Demographics: Education

Highest educational achievement	n	%
Bachelor's degree in college (4-year)	155	30.9
High school graduate	119	23.8
Some college but no degree	88	17.6
Master's degree	85	17.0
Associate degree in college (2-year)	27	5.4
Professional degree (JD, MD)	12	2.4
Less than high school degree	9	1.8
Doctoral degree	6	1.2

Table 3.

Participant Demographics: Nationality

**Nationality**	**n**	**%**
United Kingdom	165	32.9
United States of America	101	20.2
Poland	63	12.6
Portugal	57	11.4
Greece	17	3.4
Canada	13	2.6
Italy	10	2.0
Spain	10	2.0
Hungary	7	1.4
Germany	6	1.2
Netherlands	6	1.2
Estonia	5	1.0
France	4	0.8
India	4	0.8
Australia	3	0.6
South Africa	3	0.6
Austria	2	0.4
Brazil	2	0.4
Israel	2	0.4
Other*	21	4.2

Note. * Other countries reported by individual participants: Armenia, Belgium, Chile, Colombia, Czech Republic, Denmark, Finland, Iceland, Indonesia, Ireland, Latvia, Lithuania, Mexico, New Zealand, Nigeria, Romania, Singapore, Slovenia, Switzerland, Venezuela, and Viet Nam.

Table 4.

Participant Demographics: Native Language

**Native language**	**n**	**%**
English	283	56.5
Polish	64	12.8
Portuguese	57	11.4
Greek	16	3.2
Spanish	13	2.6
German	9	1.8
Italian	9	1.8
Dutch	7	1.4
Hungarian	7	1.4
Estonian	5	1.0
French	5	1.0
Hebrew	2	0.4
Other*	24	4.8

Note. * Other native languages reported by individual participants: Afrikaans, Arabic, Armenian, Bengali, Catalan, Czech, Danish, Finnish, Hindi, Icelandic, Indonesian, Kiswahili, Korean, Latvian, Lithuanian, Malayalam, Romanian, Russian, Scottish, Slovenian, Tamil, Ukrainian, Urdu, and Vietnamese.

Table 5.
Participant Demographics: Gender Identity

**Gender**	**n**	**%**
Male	257	51.3
Female	238	47.5
Prefer not to say	2	0.4
Transgender	1	0.2
Other: not specified	1	0.2
Other: genderqueer	1	0.2
Other: nonbinary	1	0.2

Tables 6-11: Cognitive Motivation and the COVID-19 Knowledge Test (Wave 1); Comparing Four Types of NC and NCC Groups

Table 6.

Descriptive Statistics of Need for Cognition Score and Need for Cognitive Closure Score (N=501)

**Scale**	**Mean**	**SD**	**Median**	**Min**	**Max**	**95% CI**
Need for Cognition	61.96	10.77	62	23	88	[61.01, 62.90]
Need for Cognitive Closure	164.15	20.08	163	107	239	[162.40, 165.91]
Order	43.19	7.6	43	17	60	[42.52, 43.85]
Predictability	33.03	6.86	33	13	48	[32.43, 33.63]
Decisiveness	25.62	6.59	25	7	42	[25.04, 26.20]
Ambiguity	38.75	5.96	39	22	54	[38.23, 39.27]
Closed Mindedness	23.56	5.08	24	8	42	[23.12, 24.01]

Table 7.

Need for Cognition and Need for Cognitive Closure scores found in the current study and previous research

	M **(SD)**	***N***
**Need for Cognition**		
Current study	61.96 (10.77)	501
Furlong (1993)	61.5 (11.4)	61
Kernis, Granneman, & Barclay (1992)	62.66 (9.17)	116
Miller, Omens, & Delvadia (1991)	62.34 (9.62)	98
Peltier & Schibrowsky (1994)	66.70 (9.37)	130
**Need for Cognitive Closure**		
Current study	3.91 (.48)	501
Djikic, Oatley, & Moldoveanu (2013)		87
Group 1	3.97 (.44)	
Group 2	3.79 (.37)	
Roets & Van Hiel (2011)*	3.77 (.74)	1584

* In this study the 41-item revision of the Need for Cognitive Closure Scale (Roets & Van Hiel, 2007) was used.

Table 8.

COVID-19 Knowledge Test True Statements Responses; Grouped on High and Low NC and NCC

Item veracity	Knowledge Test answers	NC	NCC	Mean %	95% CI
True statements (12 items)	*"I am sure is true"*	High	High	63.5	[58.6, 68.5]
		High	Low	53.8	[48.7, 59.0]
		Low	High	64.2	[59.9, 68.5]
		Low	Low	47.8	[40.5, 55.0]
	"I think this is true"	High	High	25.0	[20.7, 29.3]
		High	Low	32.3	[27.4, 37.1]
		Low	High	23.2	[19.0, 27.4]
		Low	Low	32.5	[26.7, 38.3]
	"I don't know"	High	High	6.0	[4.3, 7.8]
		High	Low	7.8	[6.0, 9.6]
		Low	High	4.6	[3.2, 6.1]
		Low	Low	12.5	[8.5, 16.5]
	*"I think this is **not** true"*	High	High	3.0	[1.7, 4.3]
		High	Low	4.6	[3.1, 6.0]
		Low	High	5.3	[3.8, 6.8]
		Low	Low	5.8	[3.9, 7.6]
	*"I am sure this is **not** true"*	High	High	2.5	[1.1, 3.8]
		High	Low	1.5	[0.8, 2.2]
		Low	High	2.6	[1.5, 3.7]
		Low	Low	1.4	[0.5, 2.4]

Note. High and Low NC were determined by a median split of the scores. High and Low NCC based on the lower and upper quantiles of scores. Importantly, the interquartile range of NCC scores were excluded. Thus, the values in this table represent 50% of the original sample. Group sizes are 61 (Hi-NC, Hi-NCC), 78 (Hi-NC,Lo-NCC), 61 (Lo-NC, Hi-NCC), and 52 (Lo-NC, Lo-NCC).

Table 9.

COVID-19 Knowledge Test False Statements Responses; Grouped on High and Low NC and NCC

Item veracity	Knowledge Test answers	NC	NCC	Mean %	95% CI
False statements (12 items)	*"I am sure is true"*	High	High	5.3	[3.4, 7.3]
		High	Low	3.8	[2.3, 5.4]
		Low	High	5.2	[3.3, 7.1]
		Low	Low	3.4	[1.3, 5.4]
	"I think this is true"	High	High	10.5	[8.2, 12.8]
		High	Low	11.6	[9.9, 13.4]
		Low	High	13.1	[10.6, 15.6]
		Low	Low	17.0	[13.6, 20.4]
	"I don't know"	High	High	11.6	[8.9, 14.3]
		High	Low	13.9	[11.3, 16.5]
		Low	High	10.8	[8.0, 13.6]
		Low	Low	20.5	[16.2, 24.8]
	*"I think this is **not** true"*	High	High	21.6	[18.1, 25.1]
		High	Low	27.5	[23.1, 31.8]
		Low	High	23.1	[19.2, 27.0]
		Low	Low	25.0	[20.6, 29.4]
	*"I am sure this is **not** true"*	High	High	51.0	[46.2, 55.7]
		High	Low	43.2	[38.8, 47.5]
		Low	High	47.8	[42.8, 52.8]
		Low	Low	34.1	[28.1, 40.2]

Note. High and Low NC were determined by a median split of the scores. High and Low NCC based on the lower and upper quantiles of scores. Importantly, the interquartile range of NCC scores were excluded. Thus, the values in this table represent 50% of the original sample. Group sizes are 61 (Hi-NC, Hi-NCC), 78 (Hi-NC,Lo-NCC), 61 (Lo-NC, Hi-NCC), and 52 (Lo-NC, Lo-NCC).

Table 10.

COVID-19 Conspiracy Statements Responses; Grouped on High and Low NC and NCC

**Item veracity**	**Knowledge Test answers**	**NC**	**NCC**	**Mean %**	**95% CI**
Conspiracy statements (7 items)	*"I am sure is true"*	High	High	0.7	[-0.3, 1.7]
		High	Low	0.0	[0.0, 0.0]
		Low	High	0.0	[0.0, 0.0]
		Low	Low	0.3	[-0.3, 0.8]
	"I think this is true"	High	High	1.4	[0.0, 2.8]
		High	Low	1.3	[-0.1, 2.7]
		Low	High	3.3	[0.5, 6.0]
		Low	Low	2.7	[0.7, 4.8]
	"I don't know"	High	High	4.4	[-0.2, 9.1]
		High	Low	13.6	[7.7, 19.4]
		Low	High	10.8	[5.3, 16.2]
		Low	Low	17.9	[10.1, 25.6]
	*"I think this is **not** true"*	High	High	12.9	[6.5, 19.3]
		High	Low	20.1	[13.6, 26.7]
		Low	High	17.6	[11.2, 23.9]
		Low	Low	28.3	[20.0, 36.6]
	*"I am sure this is **not** true"*	High	High	80.6	[72.7, 88.5]
		High	Low	65.0	[56.7, 73.4]
		Low	High	68.4	[59.5, 77.3]
		Low	Low	50.8	[40.2, 61.5]

Note. High and Low NC were determined by a median split of the scores. High and Low NCC based on the lower and upper quantiles of scores. Importantly, the interquartile range of NCC scores were excluded. Thus, the values in this table represent 50% of the original sample. Group sizes are 61 (Hi-NC, Hi-NCC), 78 (Hi-NC,Lo-NCC), 61 (Lo-NC, Hi-NCC), and 52 (Lo-NC, Lo-NCC).

Table 11.
COVID-19 Negated Conspiracy Statement Responses; Grouped on High and Low NC and NCC

Item veracity	Knowledge Test answers	NC	NCC	Mean %	95% CI
Negated conspiracy statement (1 item)	*"I am sure is true"*	High	High	49.2	[36.5, 61.8]
		High	Low	33.3	[22.8, 43.9]
		Low	High	31.1	[19.4, 42.9]
		Low	Low	17.3	[6.9, 27.7]
	"I think this is true"	High	High	27.9	[16.5, 39.2]
		High	Low	34.6	[24.0, 45.2]
		Low	High	24.6	[13.7, 35.5]
		Low	Low	30.8	[18.1, 43.4]
	"I don't know"	High	High	11.5	[3.4, 19.5]
		High	Low	12.8	[5.4, 20.3]
		Low	High	24.6	[13.7, 35.5]
		Low	Low	32.7	[19.8, 45.6]
	*"I think this is **not** true"*	High	High	3.3	[-1.2, 7.8]
		High	Low	11.5	[4.4, 18.7]
		Low	High	11.5	[3.4, 19.5]
		Low	Low	11.5	[2.8, 20.3]
	*"I am sure this is **not** true"*	High	High	8.2	[1.3, 15.1]
		High	Low	7.7	[1.7, 13.6]
		Low	High	8.2	[1.3, 15.1]
		Low	Low	7.7	[0.4, 15.0]

Note. High and Low NC were determined by a median split of the scores. High and Low NCC are based on the lower and upper quantiles of scores. Importantly, the interquartile range of NCC scores were excluded. Thus, the values in this table represent 50% of the original sample. Group sizes are 61 (Hi-NC, Hi-NCC), 78 (Hi-NC,Lo-NCC), 61 (Lo-NC, Hi-NCC), and 52 (Lo-NC, Lo-NCC).

Tables 12-16: Demographic Details of Prolific Research Participants (Wave 2)

Table 12.

Participant Demographics: Career Field (N = 326)

Category	n	%
Student	59	18.1
Information Technology	47	14.4
Unemployed	35	10.7
Marketing, Sales, and Service	27	8.3
Health Science and Health Care	24	7.4
Science, Technology, Engineering, and Mathematics	24	7.4
Education and Training	19	5.8
Arts, Audio/Video Technology, and Communications	17	5.2
Business Management and Administration	17	5.2
Finance	13	4.0
Government and Public Administration	9	2.8
Manufacturing	9	2.8
Human Services	7	2.1
Transportation, Distribution, and Logistics	7	2.1
Hospitality and Tourism	6	1.8
Law, Public Safety, Corrections, and Security	6	1.8
Agriculture, Food and Natural Resources	2	0.6
Architecture and Construction	2	0.6
Other: Retired	6	1.8
Other (please specify)	3	0.9

Note. * Other career fields reported by individual participants were blogger, educational publishing, freelancer, internet analyst, risk analyst, sex worker, sports, telecoms, and writing. Participants could choose multiple career field topics, therefore n total is larger than the sample size.

Table 13.

Participant Demographics: Education

Highest educational achievement	n	%
Bachelor's degree in college (4-year)	90	27.6
High school graduate	85	26.1
Master’s degree	66	20.3
Some college but no degree	56	17.2
Associate degree in college (2-year)	17	5.2
Professional degree (JD, MD)	7	2.2
Less than high school degree	4	1.2
Doctoral degree	1	0.3

Table 14.

Participant Demographics: Nationality

Nationality	n	%
United Kingdom	108	33.1
United States of America	53	16.3
Poland	46	14.1
Portugal	39	12.0
Greece	12	3.7
Italy	8	2.5
Canada	7	2.2
Hungary	7	2.2
Spain	7	2.2
Estonia	4	1.2
Australia	3	0.9
France	3	0.9
India	3	0.9
Netherlands	3	0.9
Brazil	2	0.6
Israel	2	0.6
South Africa	2	0.6
Other*	17	5.2

Note. * Other countries reported by individual participants: Armenia, Austria, Belgium, Chile, Czech Republic, Finland, Germany, Indonesia, Ireland, Latvia, Lithuania, Mexico, New Zealand, Nigeria, Romania, Slovenia, and Switzerland.

Table 15.

Participant Demographics: Native Language

**Native language**	**n**	**%**
English	173	53.1
Polish	46	14.1
Portuguese	39	12.0
Greek	11	3.4
Spanish	9	2.8
Hungarian	7	2.2
Italian	7	2.2
Dutch	4	1.2
Estonian	4	1.2
French	3	0.9
German	3	0.9
Hebrew	2	0.6
Other*	18	5.5

Note. * Other native languages reported by individual participants: Arabic, Armenian, Bengali, Czech, Danish, Finnish, Hindi, Indonesian, Kiswahili, Korean, Latvian, Lithuanian, Malayalam, Romanian, Russian, Scottish, Slovenian, Ukrainian, and Urdu.

Table 16.

Participant Demographics: Gender Identity

**Gender**	**n**	**%**
Male	167	51.2
Female	154	47.2
Prefer not to say	2	0.6
Transgender	1	0.3
Other: not specified	1	0.3
Other: genderqueer	1	0.3
Other: nonbinary	167	51.2

Table 17: COVID-19 Knowledge Test (Wave 2, N=326)

Table 17.

Wave 2: Descriptive Statistics COVID-19 Knowledge Test (N = 326)

Item veracity	Knowledge Test answers	Mean percentage	SD	95% CI	Min	Max
True statements (16 items)	*"I am sure this is true"*	43.5	20.9	[41.3, 45.8]	0	16/16
	"I think this is true"	29.8	17.3	[28.0 31.7]	0	14/16
	"I don't know"	14.4	10.5	[13.3, 15.5]	0	9/16
	*"I think this is **not** true"*	8.0	8.1	[7.1, 8.9]	0	10/16
	*"I am sure this is **not** true"*	4.2	8.7	[3.3, 5.2]	0	16/16
False statements (16 items)	*"I am sure this is true"*	5.1	6.4	4.4 5.8]	0	6/16
	"I think this is true"	12.5	9.4	[11.5, 13.6]	0	9/16
	"I don't know"	14.5	12.8	[13.1, 15.9]	0	10/16
	*"I think this is **not** true"*	22.4	15.7	[20.7, 24.1]	0	13/16
	*"I am sure this is **not** true"*	45.5	21.5	[43.2, 47.8]	0	16/16
Conspiracy statements (7 items)	*"I am sure this is true"*	0.9	4.7	[0.4, 1.4]	0	3/7
	"I think this is true"	3.2	10.5	[2.1, 4.4]	0	5/7
	"I don't know"	11.3	22.3	[8.8, 13.7]	0	7/7
	*"I think this is **not** true"*	19.4	26.9	[16.5, 22.3]	0	7/7
	*"I am sure this is **not** true"*	65.2	37.1	[61.1 69.2]	0	7/7
Negated conspiracy statement (1 item)	*"I am sure this is true"*	26.7	44.3	[21.9, 31.5]	Not applicable for a single item	
	"I think this is true"	26.7	44.3	[21.9, 31.5]		
	"I don't know"	22.7	42.0	[18.1, 27.3]		
	*"I think this is **not** true"*	12.9	33.6	[9.2, 16.5]		
	*"I am sure this is **not** true"*	11.0	31.4	[7.6, 14.5]		
