# Supplementary material for: Gathering, processing, and interpreting information about COVID-19
Source: Sci Rep. 2021 Mar 22;11:6569. doi: 10.1038/s41598-021-86088-3 (PMC7985139; doi:10.1038/s41598-021-86088-3)
Supplement: Supplementary file 2 — Supplementary information 2. [file 41598_2021_86088_MOESM2_ESM.pdf]

Gathering, Processing, and Interpreting Information About COVID-19

Arnout B. Boot, Anita Eerland, Joran Jongerling, Peter P.J.L. Verkoeijen, and Rolf A. Zwaan

Appendix B

Supplementary Figures

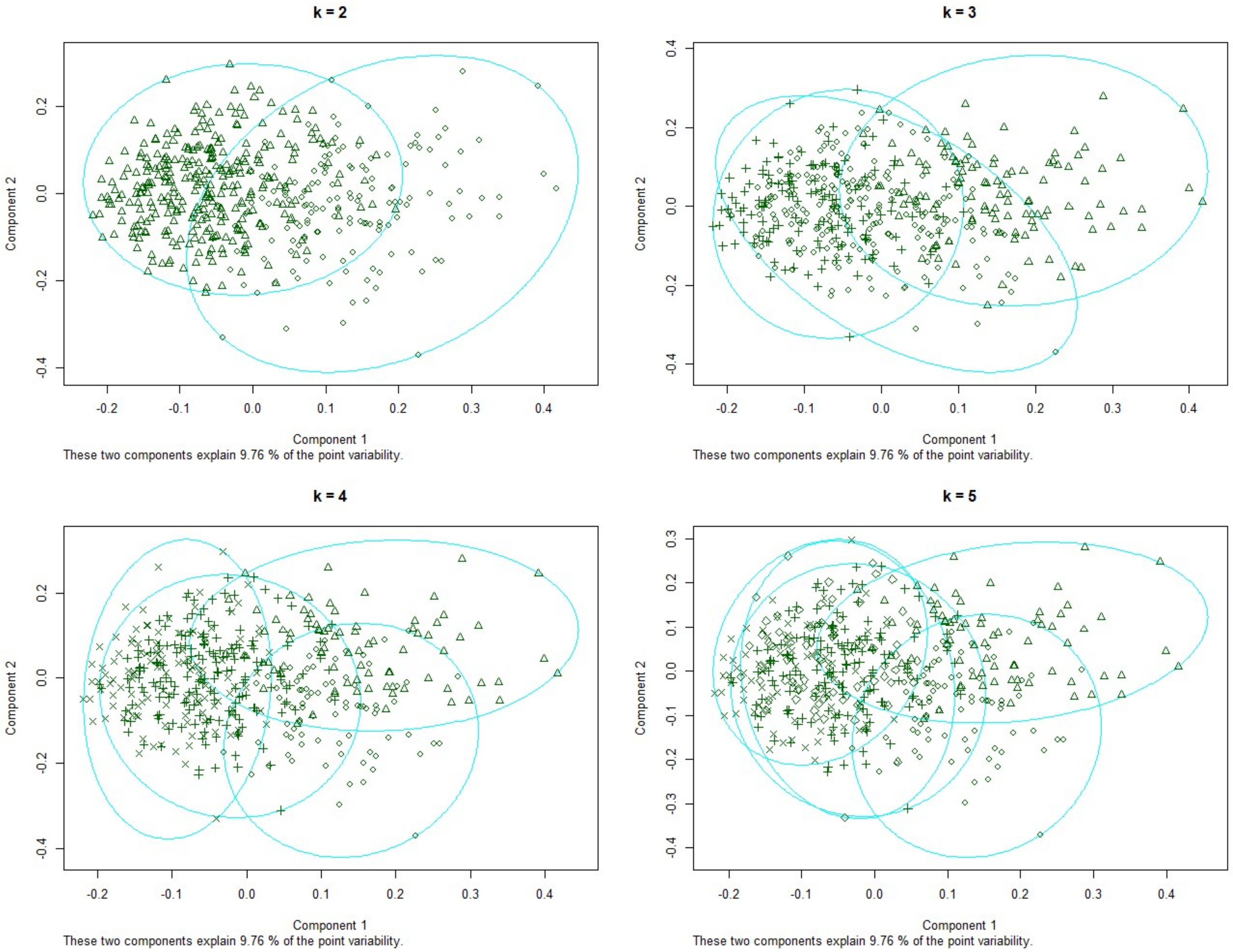

Figure 1. **Cluster Analysis.** Cluster plots for 2, 3, 4, and 5 clusters. The 32 items of the COVID-19 Knowledge test (24 items), Conspiracy Rejection (8 items), NCC scores, NC scores, time spent on social media, frequency of checking COVID-19 updates, deaths per million in a respondent's country (on March 27th), measuring the use of sources consulted for information on COVID-19 (14 items), and reasons for consulting news sources (5 items). Given that many of these variables have an ordinal measurement level, the cluster analysis was based on the general dissimilarity coefficient of Gower (1971), calculated using the *daisy* function from the *cluster* R-package (Maechler et al., 2019). The resulting dissimilarity matrix was subsequently used as input for analyses in which the data was partitioned into *k* (*k* = 1, 2, 3, 4, and 5) clusters "around medoids", a more robust version of k-means clustering. All analyses used the *pam* function from the *cluster* package (Maechler et al., 2019).

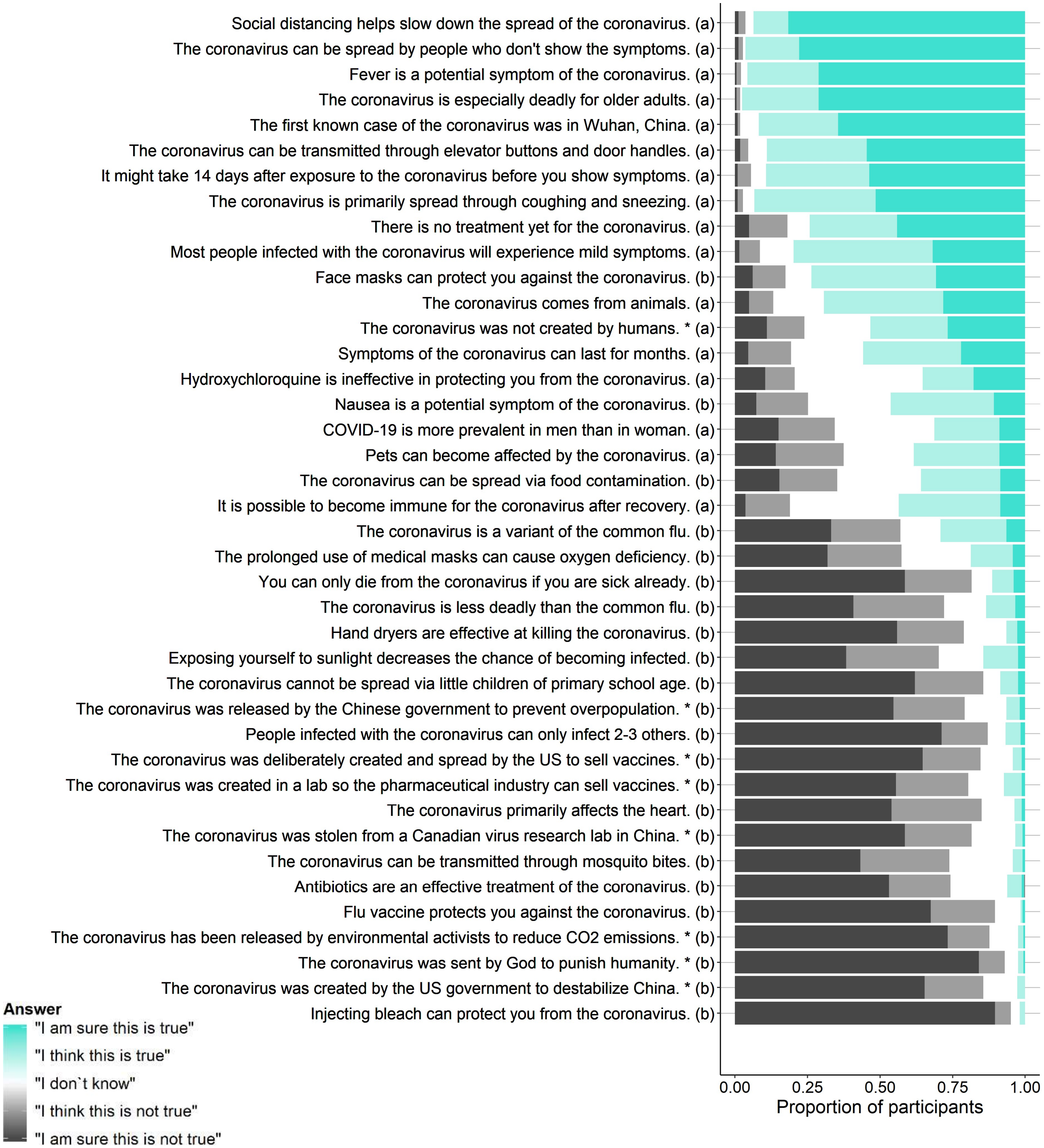

Figure 2. **COVID-19 Knowledge Test Wave 2** (N = 326). True (a), false (b) and conspiracy (*) statements.

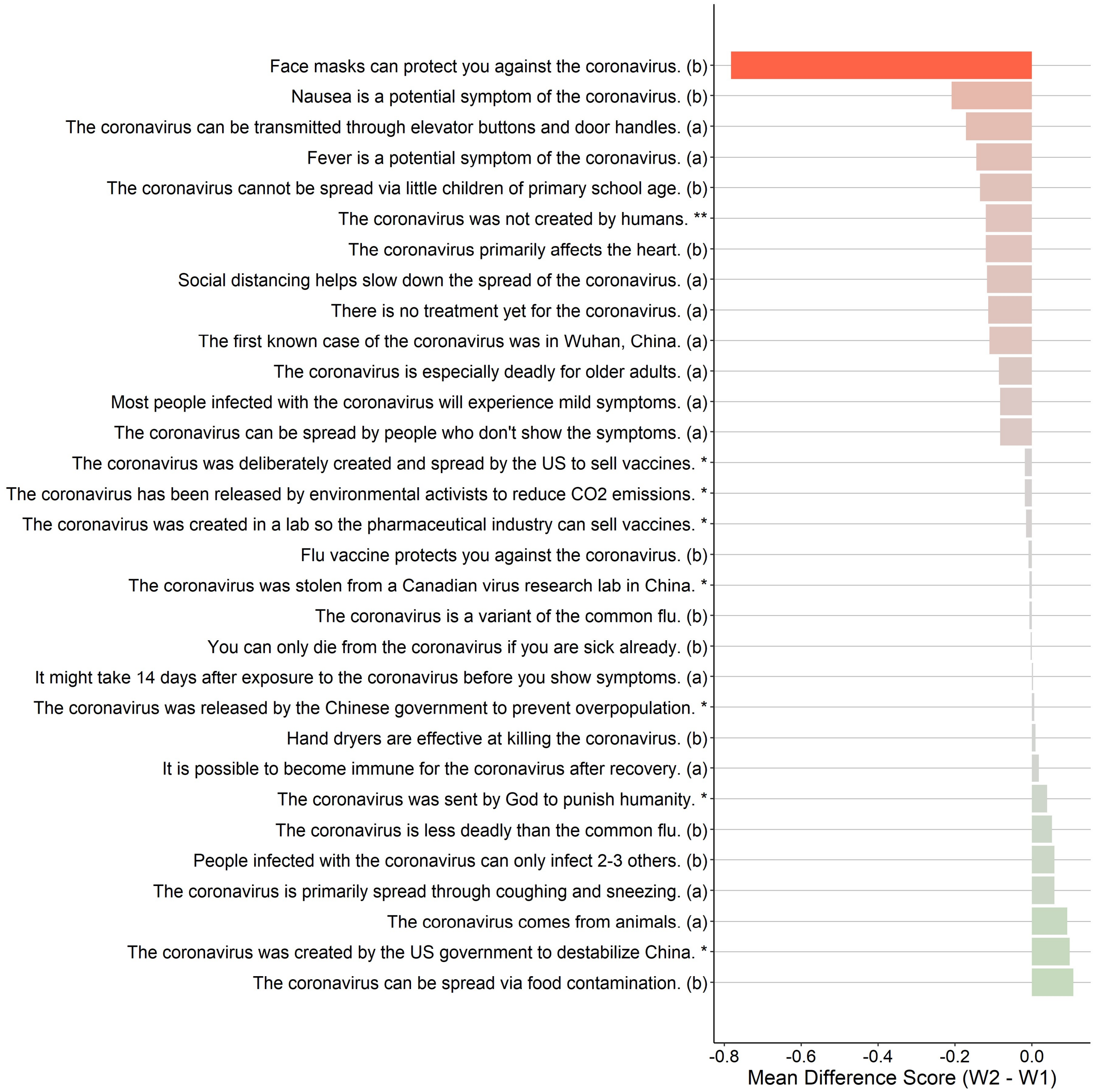

Figure 3. **Difference Scores COVID-19 Knowledge Test**. Mean difference scores between Wave 1 and Wave 2 for true (a), false (b) and conspiracy (*) statements in the COVID 19 Knowledge Test. Negative values indicate lowered accuracy.

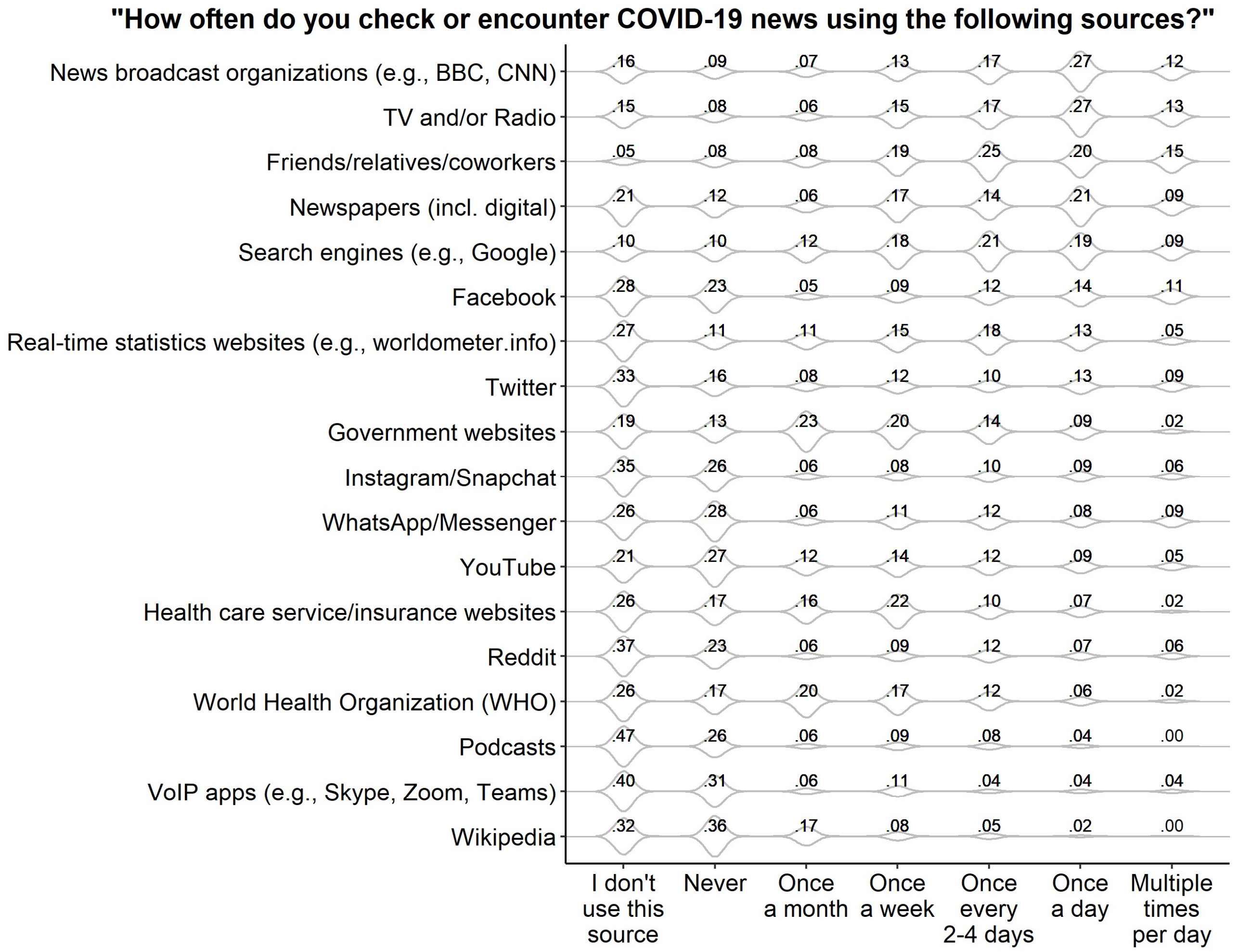

Figure 4. **Media Use Wave 2.** Frequency by which different sources are consulted for COVID-19 information.

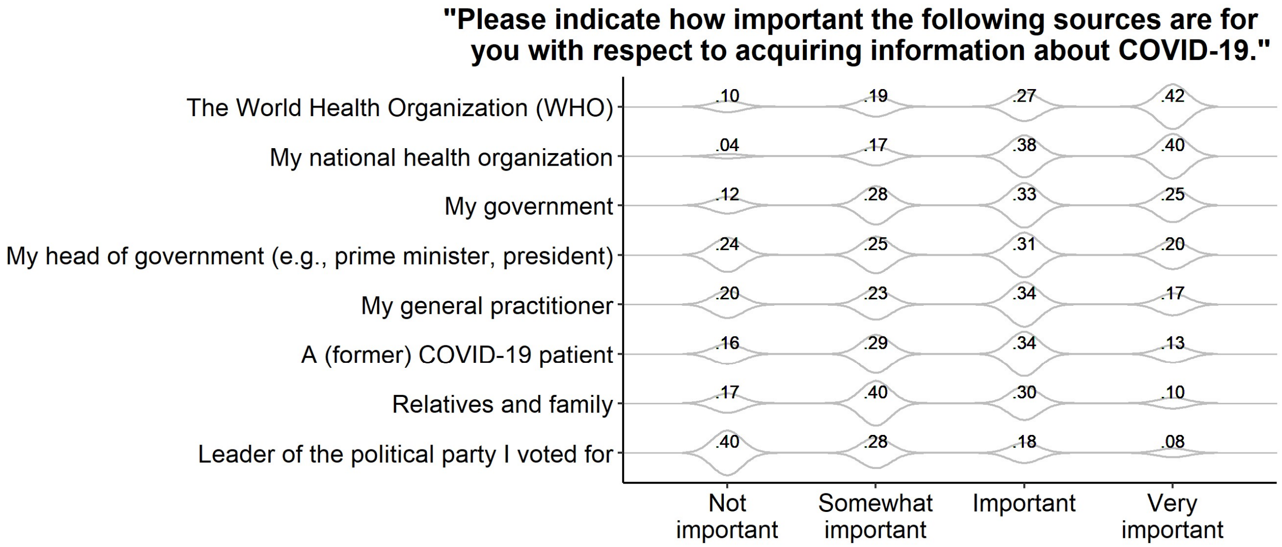

Figure 5. **Respected Sources Wave 2**. The perceived importance of different sources for COVID-19 information.

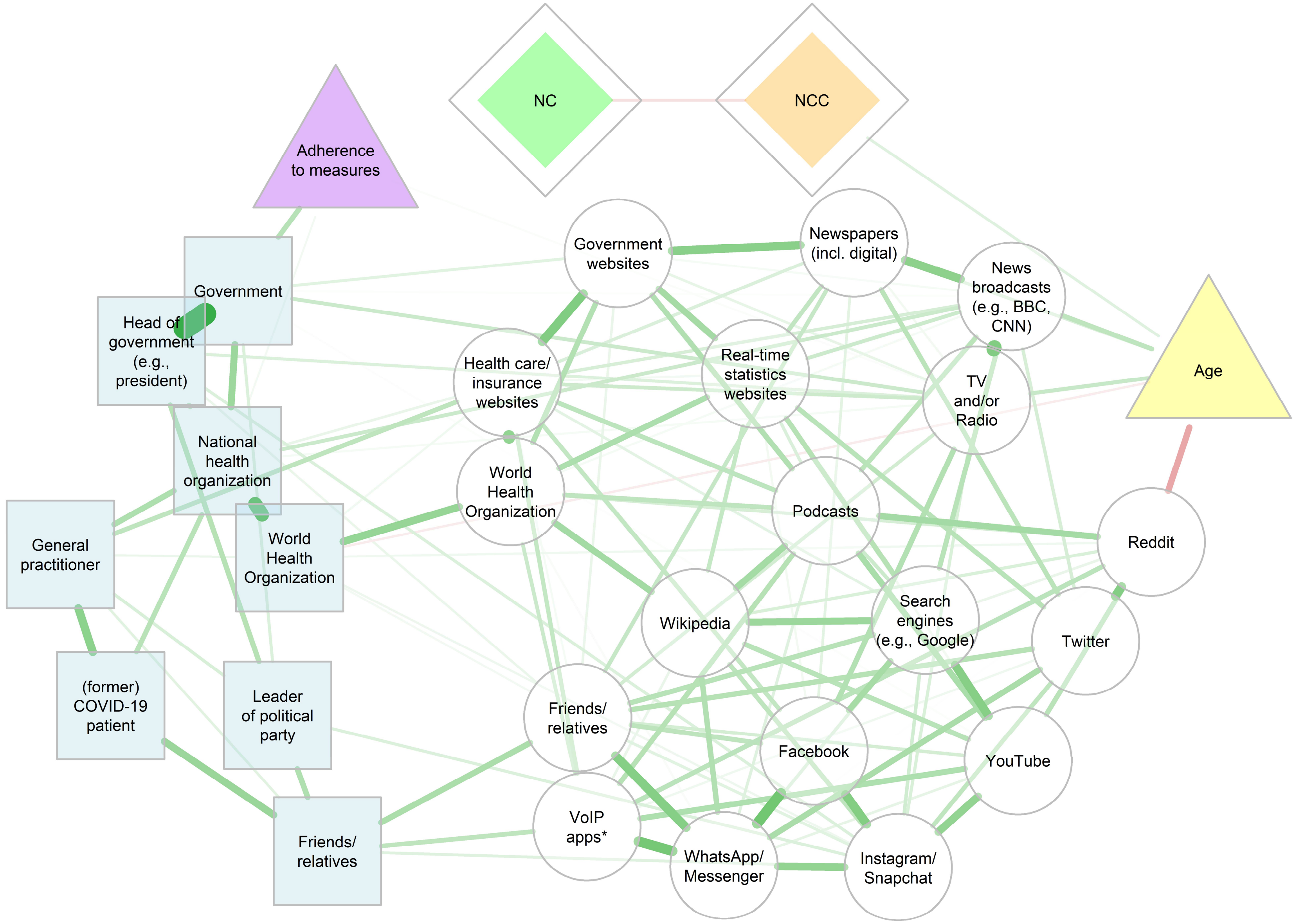

Figure 6. **Network Analysis Wave 2**. Network analysis displaying the relations between NC, NCC, the perceived importance of different sources (blue squares), sources that are used to acquire information about COVID-19 (white circles), adherence to (government-imposed) measures, and age.

References (Appendix B)

Gower, J. C., & Legendre, P. (1986). Metric and Euclidean properties of dissimilarity coefficients. *Journal of classification*, *3*(1), 5-48.

Maechler, M., Rousseeuw, P., Struyf, A., Hubert, M., & Hornik, K. (2019). cluster: Cluster Analysis Basics and Extensions. R package version 2.1.0.
