# Supplementary material for: Gathering, processing, and interpreting information about COVID-19
Source: Sci Rep. 2021 Mar 22;11:6569. doi: 10.1038/s41598-021-86088-3 (PMC7985139; doi:10.1038/s41598-021-86088-3)
Supplement: Supplementary file 3 — Supplementary information 3. [file 41598_2021_86088_MOESM3_ESM.pdf]

Gathering, Processing, and Interpreting Information About COVID-19

Arnout B. Boot, Anita Eerland, Joran Jongerling, Peter P.J.L. Verkoeijen, and Rolf A. Zwaan

Appendix C

COVID-19 Knowledge Test

Conspiracy statement evaluations (pp. 1-2)

Participants were asked:" What is your current view on the following statements about the coronavirus? (Try to answer promptly based on your first instinct)".

Values in the bars represent percentages of groups (Group sizes: $n_{Hi\text{-}NCC,\ Hi\text{-}NC} = 61$; $n_{Hi\text{-}NCC,\ Lo\text{-}NC} = 61$; $n_{Lo\text{-}NCC,\ Hi\text{-}NC} = 78$; $n_{Lo\text{-}NCC,\ Lo\text{-}NC} = 52$). * The asterisks in the bars indicate only one participant gave this answer.

"I am sure this is true"
"I think this is true"
"I don`t know"
"I think this is not true"
"I am sure this is not true"

The coronavirus was released by the Chinese government to prevent overpopulation.

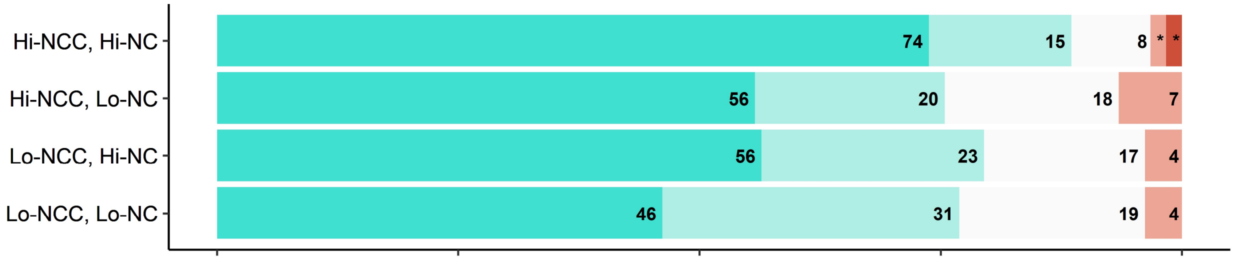

The coronavirus was created in a lab so the pharmaceutical industry can sell vaccines.

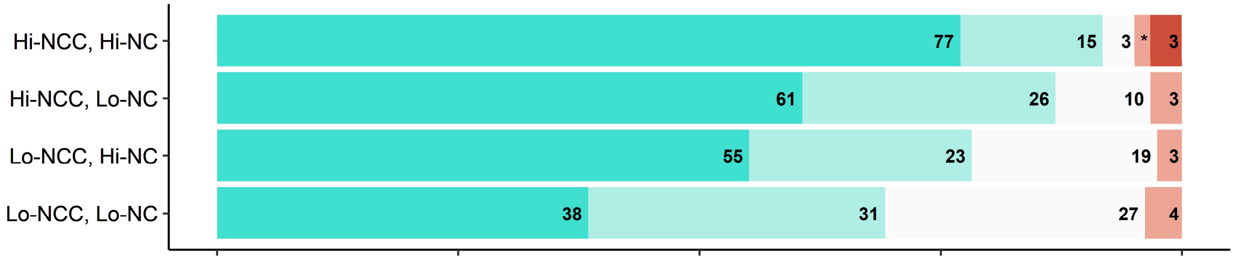

The coronavirus was sent by God to punish humanity.

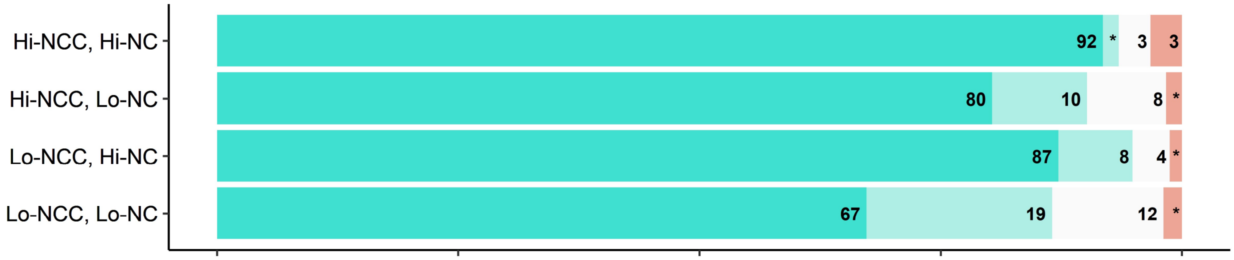

The coronavirus has been released by environmental activists to reduce CO2 emissions.

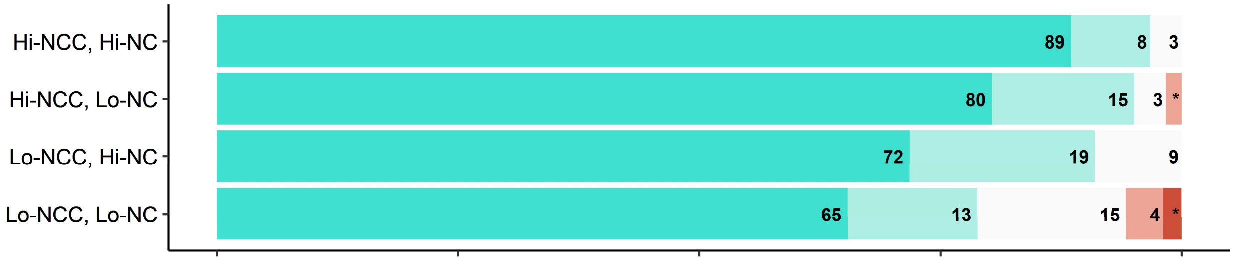

The coronavirus was created by the US government to destabilize China.

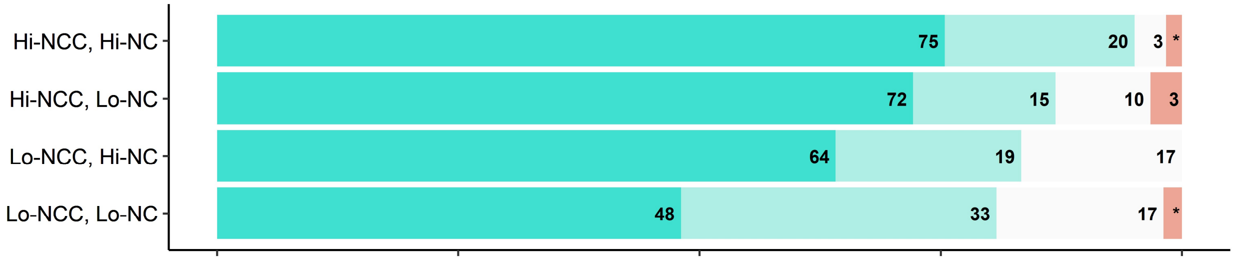

The coronavirus was deliberately created and spread by the US to sell vaccines.

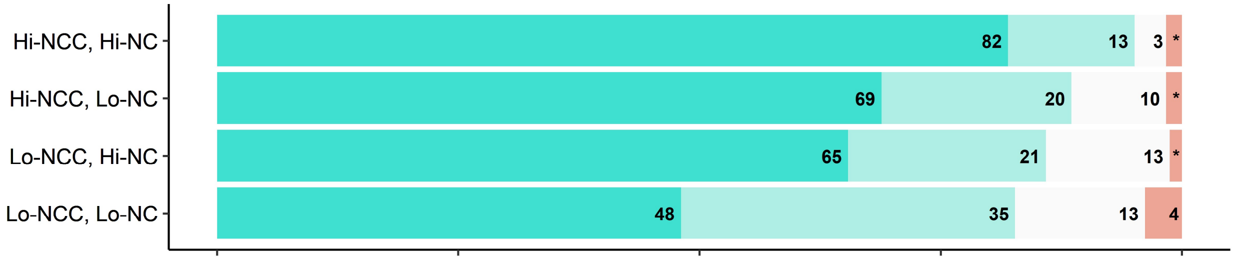

The coronavirus was stolen from a Canadian virus research lab in China.

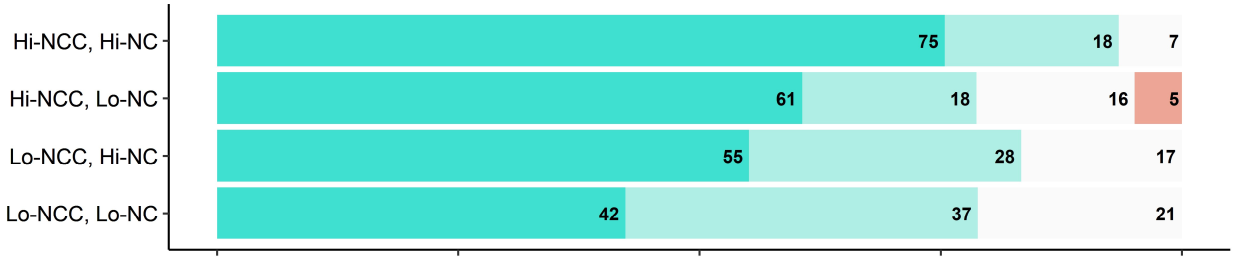

The coronavirus was not created by humans.*

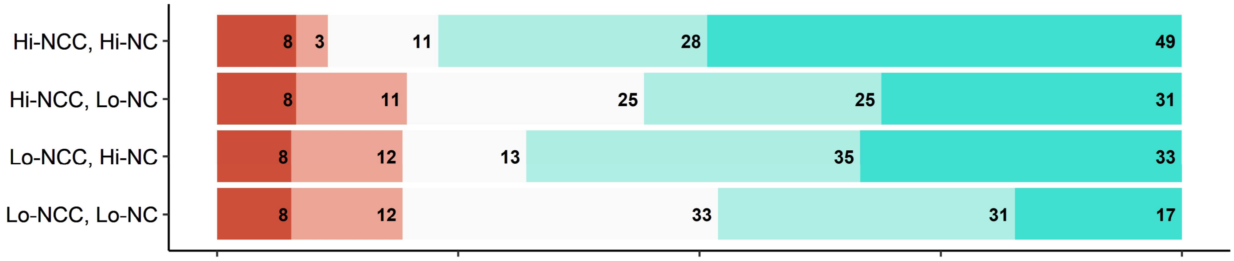

Note. This statement contains a negation; color codes are reversed: ‘this is not true’ in red and ‘this is true” in blue

.

***True* statement evaluations (pp. 3-7)**

Participants were asked:” What is your current view on the following statements about the coronavirus? (Try to answer promptly based on your first instinct)”.

Values in the bars represent percentages of groups (Group sizes: $n_{Hi\text{-}NCC, Hi\text{-}NC}$ = 61; $n_{Hi\text{-}NCC, Lo\text{-}NC}$ = 61; $n_{Lo\text{-}NCC, Hi\text{-}NC}$ = 78; $n_{Lo\text{-}NCC, Lo\text{-}NC}$ = 52). * The asterisks in the bars indicate only one participant gave this answer.

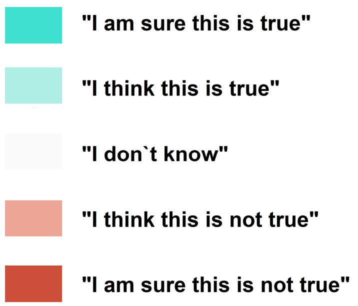

The first known case of the coronavirus was in Wuhan, China.

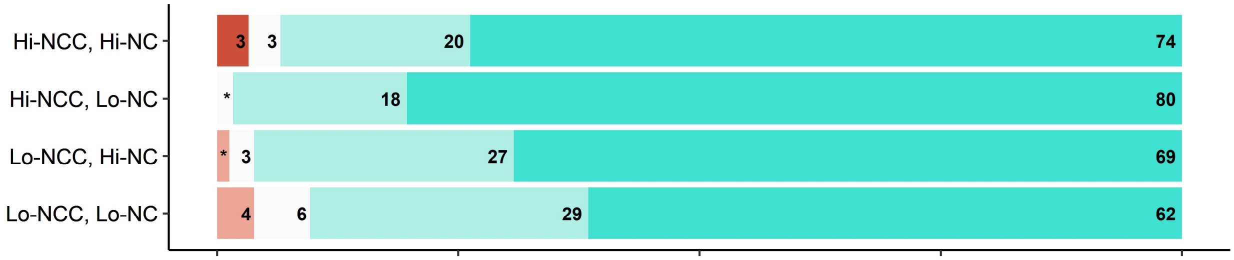

The coronavirus is primarily spread through coughing and sneezing.

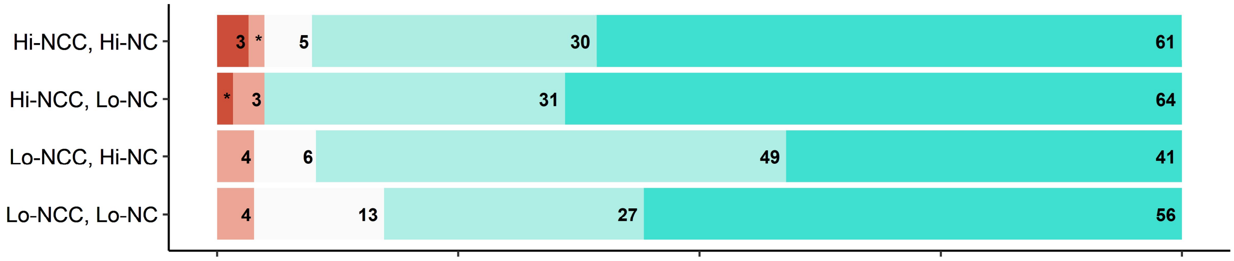

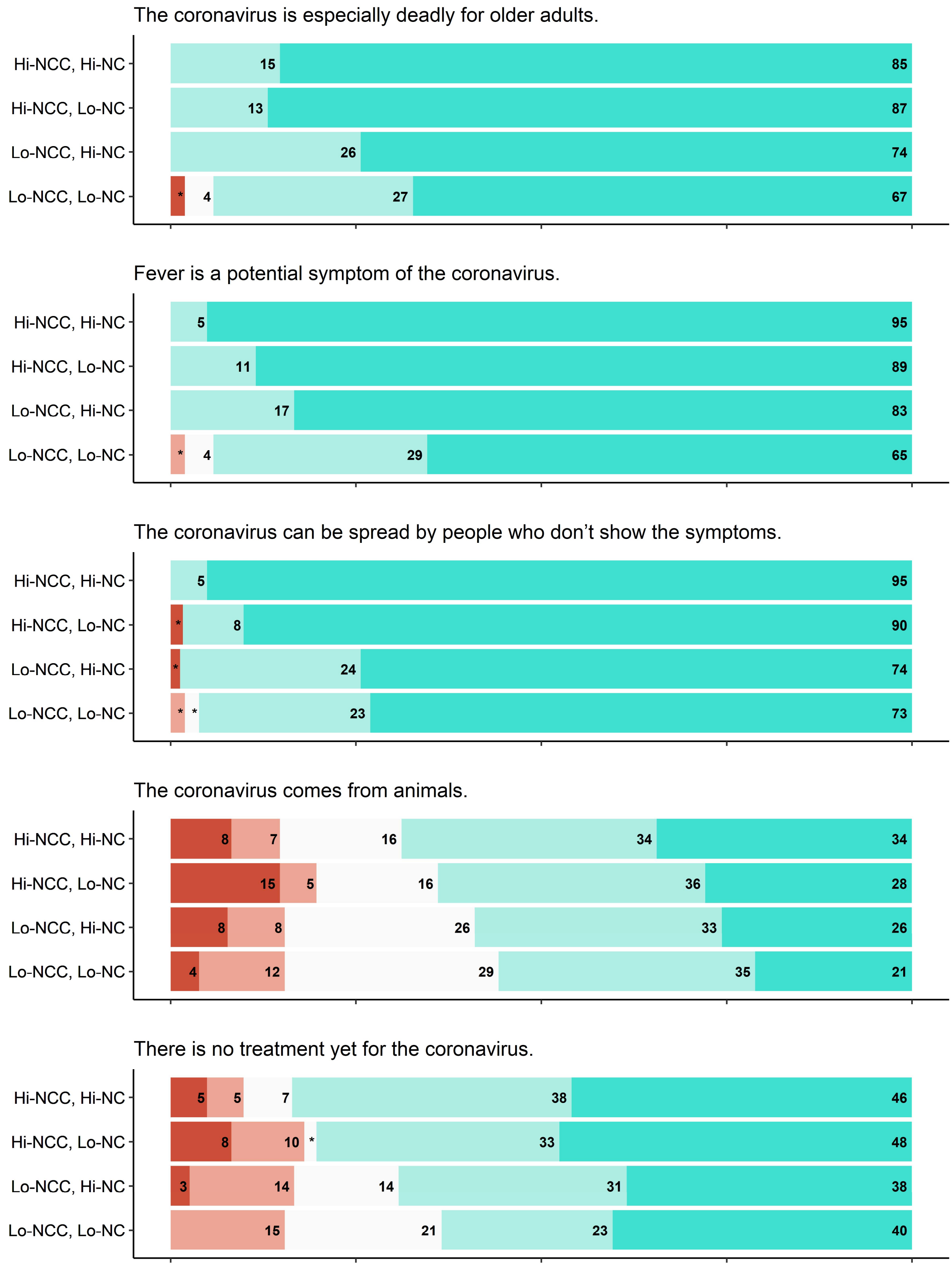
The coronavirus is especially deadly for older adults.
Hi-NCC, Hi-NC
15
85
Hi-NCC, Lo-NC
13
87
Lo-NCC, Hi-NC
26
74
Lo-NCC, Lo-NC
*
4
27
67
Fever is a potential symptom of the coronavirus.
Hi-NCC, Hi-NC
5
95
Hi-NCC, Lo-NC
11
89
Lo-NCC, Hi-NC
17
83
Lo-NCC, Lo-NC
*
4
29
65
The coronavirus can be spread by people who don't show the symptoms.
Hi-NCC, Hi-NC
5
95
Hi-NCC, Lo-NC
*
8
90
Lo-NCC, Hi-NC
*
24
74
Lo-NCC, Lo-NC
*
*
23
73
The coronavirus comes from animals.
Hi-NCC, Hi-NC
8
7
16
34
34
Hi-NCC, Lo-NC
15
5
16
36
28
Lo-NCC, Hi-NC
8
8
26
33
26
Lo-NCC, Lo-NC
4
12
29
35
21
There is no treatment yet for the coronavirus.
Hi-NCC, Hi-NC
5
5
7
38
46
Hi-NCC, Lo-NC
8
10
*
33
48
Lo-NCC, Hi-NC
3
14
14
31
38
Lo-NCC, Lo-NC
15
21
23
40

It might take 14 days after exposure to the coronavirus before you show symptoms.

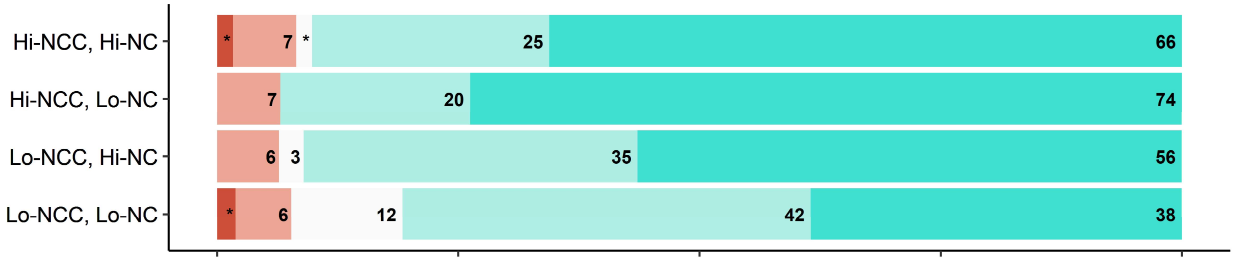

Social distancing helps slow down the spread of the coronavirus.

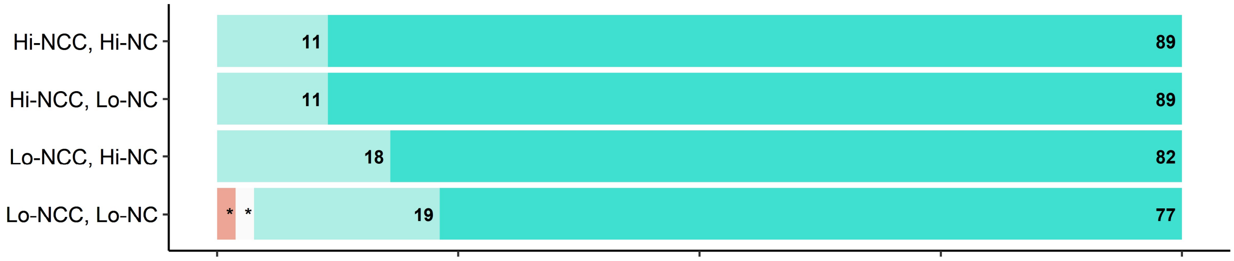

It is possible to become immune for the coronavirus after recovery.

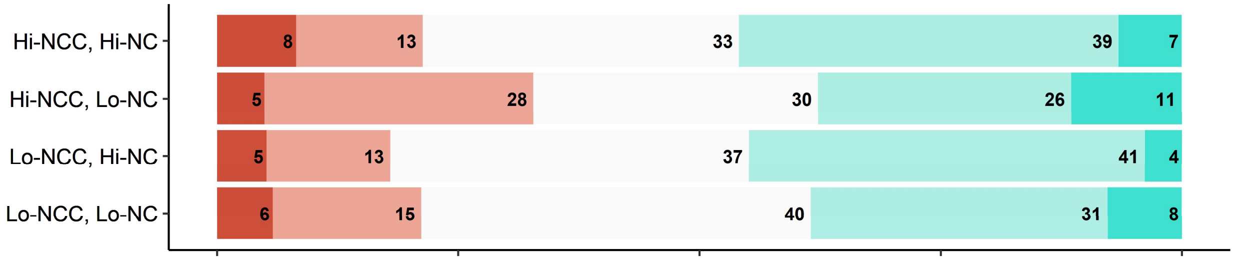

Most people infected with the coronavirus will experience mild symptoms.

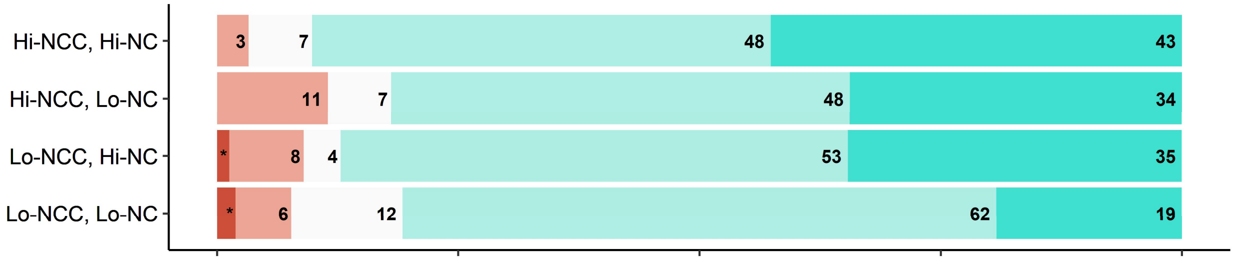

The coronavirus can be transmitted through elevator buttons and door handles.

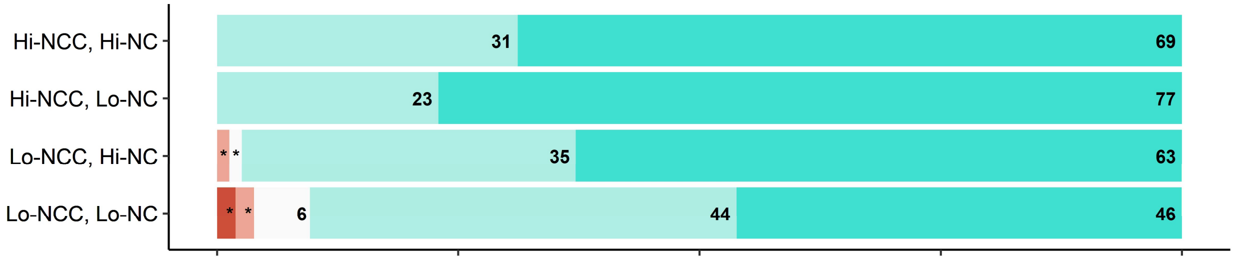

False statement evaluations (pp. 6-8)

Participants were asked:" What is your current view on the following statements about the coronavirus? (Try to answer promptly based on your first instinct)".

Values in the bars represent percentages of groups (Group sizes: $n_{Hi\text{-}NCC,\ Hi\text{-}NC} = 61$; $n_{Hi\text{-}NCC,\ Lo\text{-}NC} = 61$; $n_{Lo\text{-}NCC,\ Hi\text{-}NC} = 78$; $n_{Lo\text{-}NCC,\ Lo\text{-}NC} = 52$). * The asterisks in the bars indicate only one participant gave this answer.

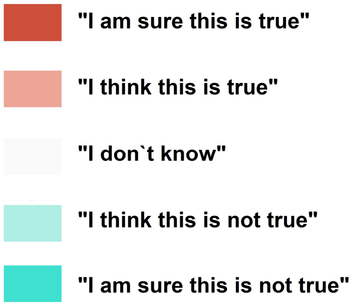

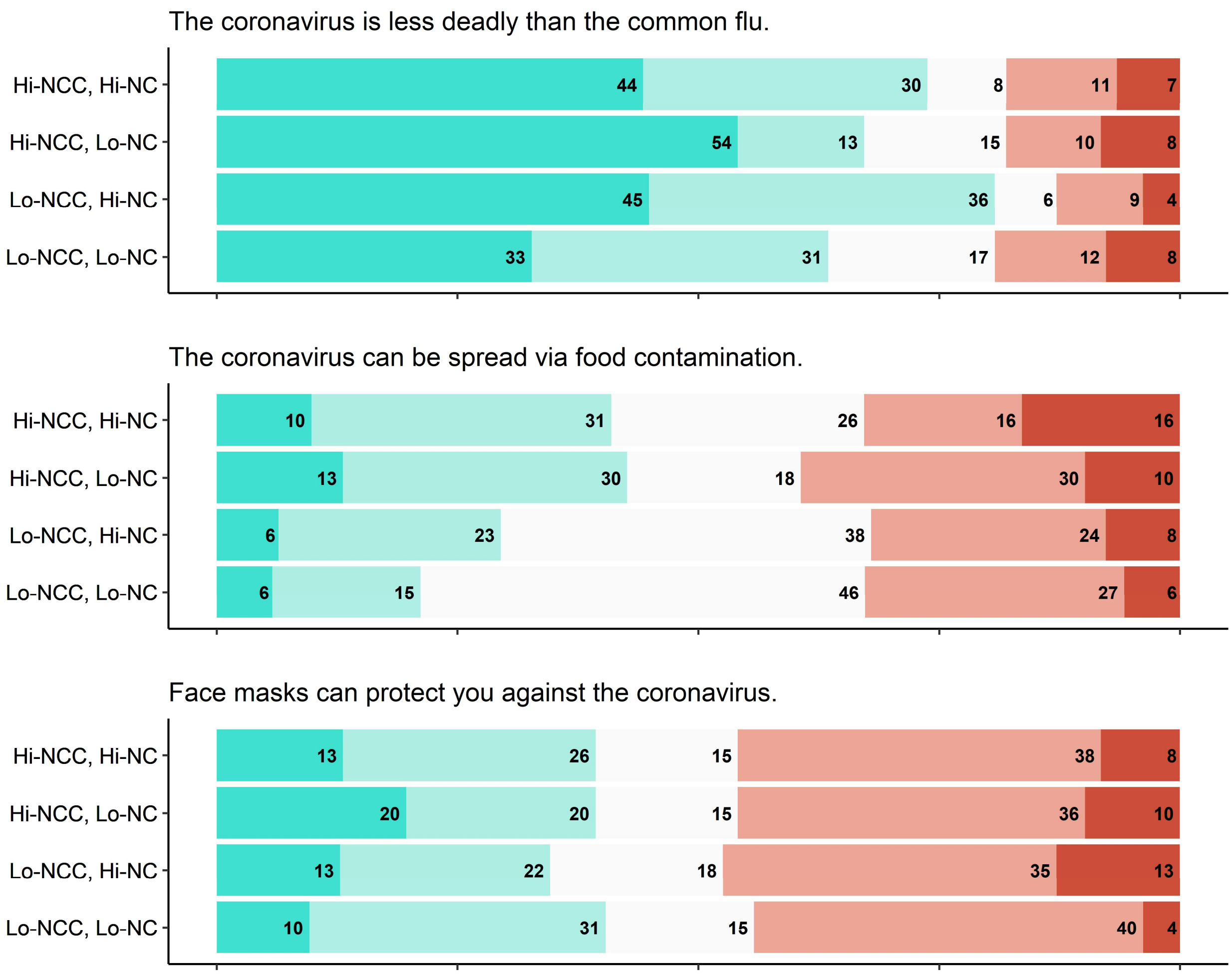

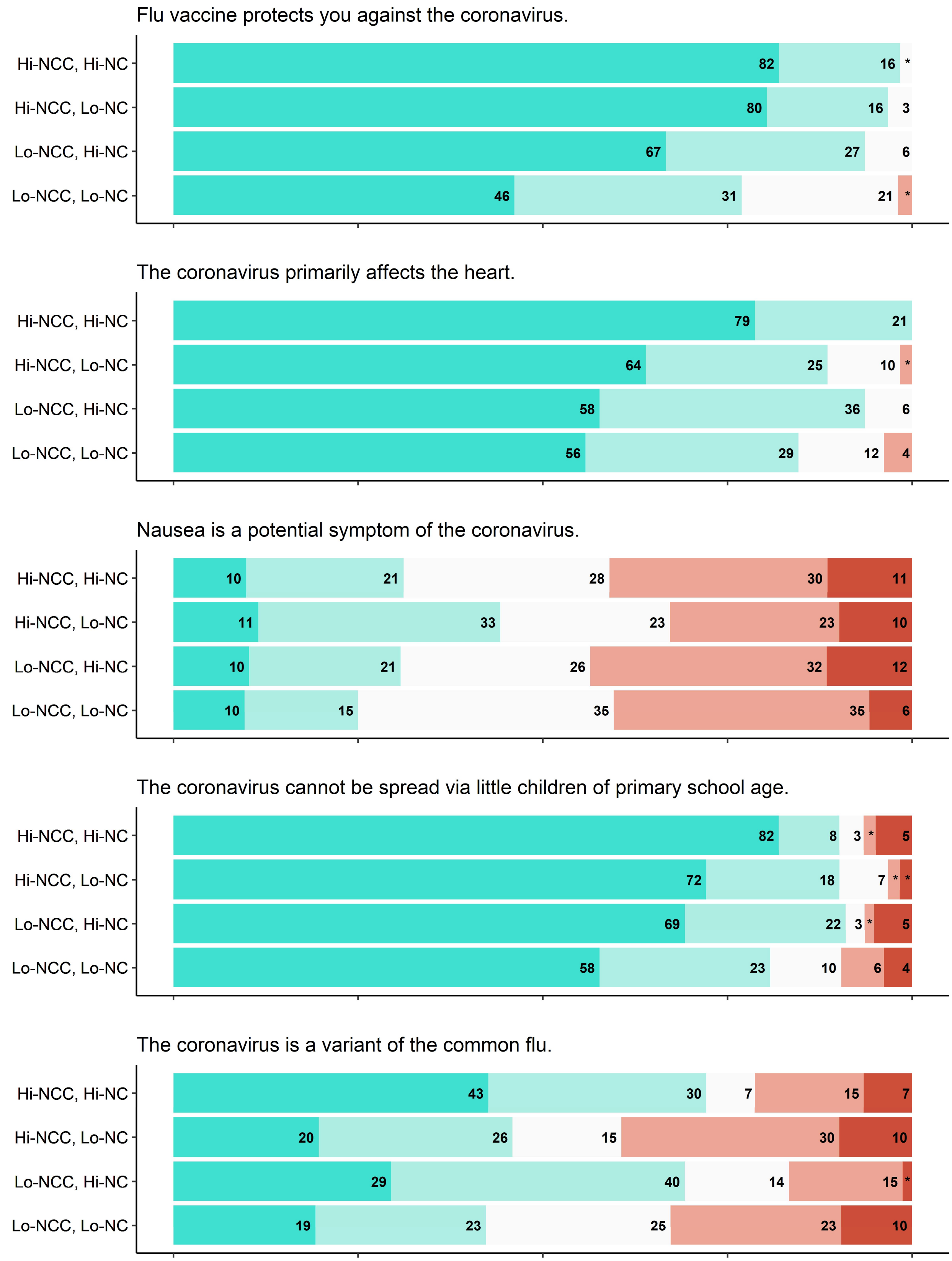
Flu vaccine protects you against the coronavirus.
Hi-NCC, Hi-NC
82
16
*
Hi-NCC, Lo-NC
80
16
3
Lo-NCC, Hi-NC
67
27
6
Lo-NCC, Lo-NC
46
31
21
*
The coronavirus primarily affects the heart.
Hi-NCC, Hi-NC
79
21
Hi-NCC, Lo-NC
64
25
10
*
Lo-NCC, Hi-NC
58
36
6
Lo-NCC, Lo-NC
56
29
12
4
Nausea is a potential symptom of the coronavirus.
Hi-NCC, Hi-NC
10
21
28
30
11
Hi-NCC, Lo-NC
11
33
23
23
10
Lo-NCC, Hi-NC
10
21
26
32
12
Lo-NCC, Lo-NC
10
15
35
35
6
The coronavirus cannot be spread via little children of primary school age.
Hi-NCC, Hi-NC
82
8
3
*
5
Hi-NCC, Lo-NC
72
18
7
*
*
Lo-NCC, Hi-NC
69
22
3
*
5
Lo-NCC, Lo-NC
58
23
10
6
4
The coronavirus is a variant of the common flu.
Hi-NCC, Hi-NC
43
30
7
15
7
Hi-NCC, Lo-NC
20
26
15
30
10
Lo-NCC, Hi-NC
29
40
14
15
*
Lo-NCC, Lo-NC
19
23
25
23
10

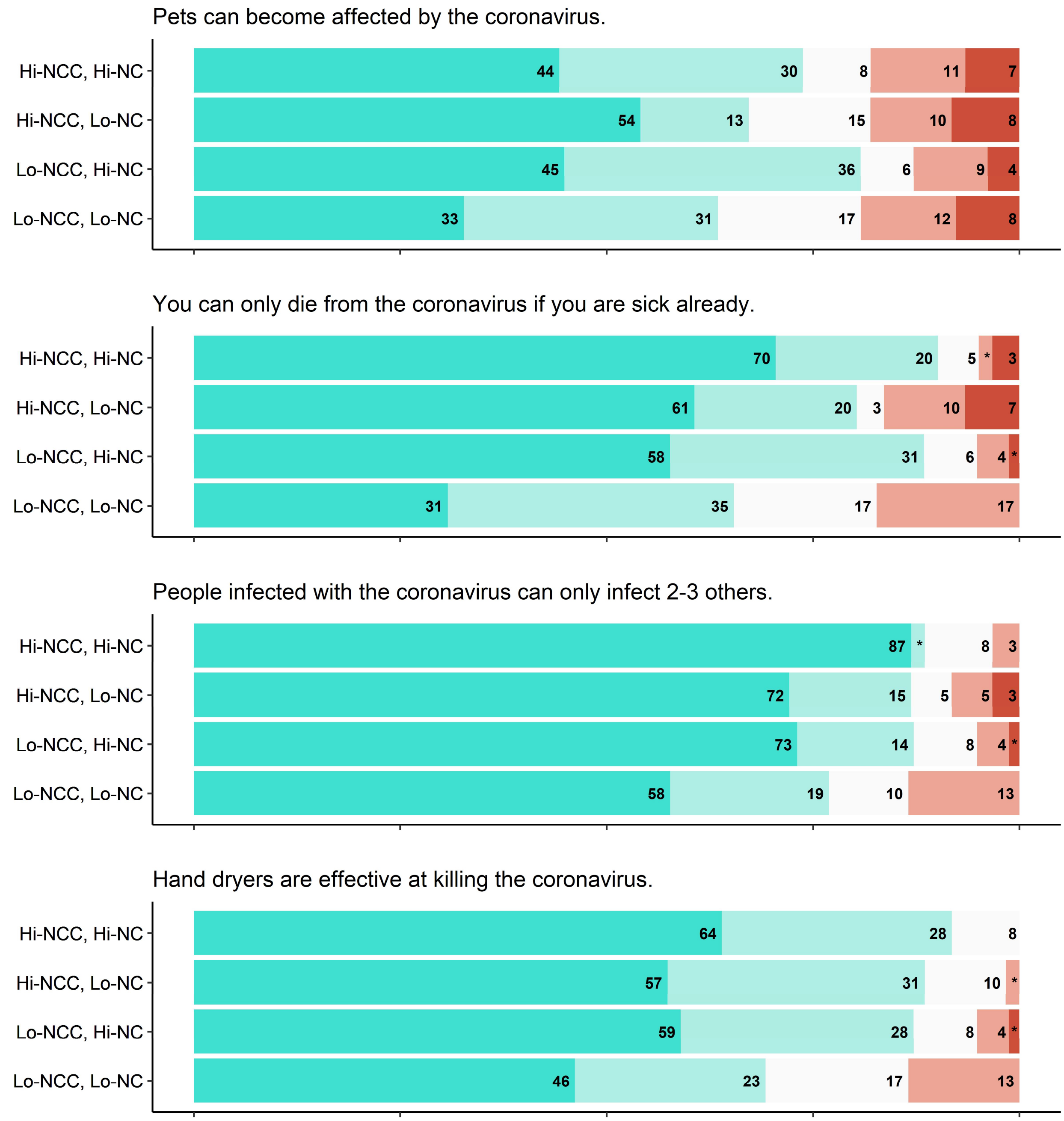
Pets can become affected by the coronavirus.
Hi-NCC, Hi-NC
44 30 8 11 7
Hi-NCC, Lo-NC
54 13 15 10 8
Lo-NCC, Hi-NC
45 36 6 9 4
Lo-NCC, Lo-NC
33 31 17 12 8
You can only die from the coronavirus if you are sick already.
Hi-NCC, Hi-NC
70 20 5 * 3
Hi-NCC, Lo-NC
61 20 3 10 7
Lo-NCC, Hi-NC
58 31 6 4 *
Lo-NCC, Lo-NC
31 35 17 17
People infected with the coronavirus can only infect 2-3 others.
Hi-NCC, Hi-NC
87 * 8 3
Hi-NCC, Lo-NC
72 15 5 5 3
Lo-NCC, Hi-NC
73 14 8 4 *
Lo-NCC, Lo-NC
58 19 10 13
Hand dryers are effective at killing the coronavirus.
Hi-NCC, Hi-NC
64 28 8
Hi-NCC, Lo-NC
57 31 10 *
Lo-NCC, Hi-NC
59 28 8 4 *
Lo-NCC, Lo-NC
46 23 17 13
