# Supplementary material for: Gathering, processing, and interpreting information about COVID-19
Source: Sci Rep. 2021 Mar 22;11:6569. doi: 10.1038/s41598-021-86088-3 (PMC7985139; doi:10.1038/s41598-021-86088-3)
Supplement: Supplementary file 4 — Supplementary information 4. [file 41598_2021_86088_MOESM4_ESM.pdf]

Gathering, Processing, and Interpreting Information About COVID-19

Arnout B. Boot, Anita Eerland, Joran Jongerling, Peter P.J.L. Verkoeijen, and Rolf A. Zwaan

Appendix D

Supplementary examples 'adherence to government-imposed measures'

We investigated the respondents' adherence to government-imposed measures and asked for an explanation of their reported adherence. Most respondents (48.5%) reported to 'always' follow the measures. Typical explanations for their compliant behaviour were:

Example 1. *"I've been following the measures imposed by my government, because it's important to stop the spread of the virus, and we all should do our job to avoid the negative impact of this disease."*

Example 2. *"It is very important to follow the lockdown measures in order for coronavirus cases to slow down."*

Example 3. *"I wash my hands, use gel when out and about and wear a face mask. I stay at home and only leave for essential journeys."*

The second largest group (37.1%) reported to 'often' follow the measures. Typical explanations for their behaviour were:

Example 4. *"I tend to stick to the rules of the restrictions, but occasionally 'bend' them slightly to do things that I enjoy doing. I feel most other people are doing this to a much greater extent than I am."*

Example 5. *"I try to follow them all the time but it's difficult when I encounter someone else who doesn't and stands too close to me for example."*

Some respondents reported to comply with initial measures against COVID-19, but they did not agree with the successive relaxations of these measures:

Example 6. *"I complied with all rules and restrictions but I have not changed any of my behaviours since March, by that I mean I am not relaxing my behaviour as much as my government has said I can, as I do not believe it is safe to do so."*

A smaller group (9.8%) reported to 'sometimes' follow the news. Typical explanations for their low compliance were:

Example 7. *"I do not believe that virus exist in the dimensions they say, I comply with basic hygiene rules such as washing my hands and having a respiratory label, but I do not stay at home isolated or miss my family."*

Example 8. *"There were some decisions that are taken on the base of fear and population control, and although is necessary, I don't like it. And it also felt like our liberty was being paused."*

Example 9. *"The environment that I work in makes it difficult to socially distance. The rules for this seem to have been disregarded."*

Other respondents within this group were less compliant because their governments were not actively imposing adequate measures:

Example 10. *"If referring to our federal government, I don't feel like there has been explicit guidance coming from the top. So, I try to follow what I see and hear from medical sources and from other countries that appear to have successfully contained the virus for the moment."*

The smallest group reported to either 'rarely' (2.8%) or to never (1.8%) follow the measures. Typical explanations for their lack of compliance were:

Example 11. *"The government didn't really seem like they knew what they were doing at the start. Measures were implemented in the course of the past few months that were more totalitarian than to do with virus prevention."*

Example 12. *"My government did the exact opposite of what every health organization said to do. I'm not following them off a cliff."*

Cases of low adherence can mainly be attributed to inadequate government activity. For example, from the 15 respondents who reported to either 'never' or 'rarely' follow the measures 10 respondents (67%) explained this by a lack of government-imposed guidelines. Likewise, from the 32 respondents who reported to 'sometimes' follow the measures 11 respondents (34%) explained this by an inadequacy or lack of government-imposed guidelines. Only four respondents (1.2%) appeared to be noncompliant (i.e., 'never' or 'rarely' follow measures) for other reasons than inadequacy of their government. Their explanations:

Example 13. *"I just don't want to take part in it, it's all utter nonsense and lies, everything. Some things I have to do because we all need food, but I will avoid queues, masks, public transport (because of masks), tests, apps, digital passports and especially vaccines. I'm Out!"*

Example 14. *Everyone wearing masks here are not covering their nose, so wearing it or not doesn't make a difference. Moreover, I believe masks are made to be worn in order to destroy humans' natural bacteria flora which strengthens itself by having facial and hand contact with others.*

Example 15. *"There is no COVID-19."*

Example 16. *"I don't watch the news or listen to it on the radio. I think it's a conspiracy."*

EXPLANATIONS FOR ADHERENCE TO GOVERNMENT-IMPOSED MEASURES

"To what degree do you follow the measures your government took in reaction to COVID-19? Please explain your answer in the text box below."

Participants who ***always*** follow measures

Reference nr.	Response
1	it is better to read and take as much as safety because its for our well being and good health.
2	I always wear a facemask and gloves when going outside, and I limit how often I go outside to limit exposure to the virus.
3	I follow all restrictions regarding protecting myself from COVID-19
4	i follow all the measures
5	Have abided by all requirements to socially distance and stay at home
6	I comply to the rules
7	We have to have common sense to understand that everything is done for our own safety
8	I always follow the measures even if a lot of people don't
9	If goverment tells us people that we must wear masks I wear them everytime. Even if they are not obligatory now I wear them too because I worry that I am less immune than others.
10	I always follow it
11	They still didn't do enough or act quickly enough. I follow their vague advice as best I can.
12	Always use facemask when in public and stay at home as long as possible

13	I always wear a mask, wash my hands, wipe down products such as shopping or orders and keep social distancing.
14	I respected lockdown rules - since it started, I have only been out for groceries. I wear a mask, leave my partner and child in the car and make it quick. I cancelled all plans, am working from home (with a brief stint of being furloughed), I am trying my best to homeschool my daughter/care for her while school is closed. I haven't seen my parents or my friends for months.<br><br>My daughter's school is open again now, but we have made the decision not to send her back until September. I will not be going to any of the newly opened 'non-essential' shops or places like pubs as I still think it's too risky.
15	Even though it's a drag I don't mean to cause any trouble for anyone, especially to myself.
16	I followed all recommendations issued by our government (limiting being outside to necessary matters, wearing face masks in public, switching to home-office, homeschooling children)
17	I am making sure to stay in the house as much as possible and only go out to get few once every few weeks.
18	The government will always try to do what is best for everyone
19	I have rarely left home, only going out for groceries or for exercise. I have maintained a 2 metre gap between me and others wherever possible. I have avoided public transport. I have washed my hands regularly and wiped-down surfaces with disinfectant.
20	I wear a mask when in public, practice social distancing, I don't go outside unless necessary
21	I use security measures but I certainly do not approve of what the government has done, too many deaths before acting
22	I have followed Govt guidelines as requested in terms of hand washing, social distancing etc
23	I wanted to keep my family and myself safe.

24	I follow the recommendations to wear masks in public places, I don't pay in shops in cash and with my card, I avoid large groups of people, I don't attend mass events, I work remotely at home.
25	I would personally take care either way.
26	Followed the guidelines, stayed at home
27	I stayed inside and i am very carefull folowing protocol.
28	I stay in as much as I can, I only go out to food shop, I dont touch my face and I wash my hands as much as possible.
29	I always do what my government says to try to protect the people and help not spread the virus. *(note. This comment was corrected, because the participant commented via Prolific: "In the last question I meant to help not spread the virus")*
30	I practice all the measures recommended , washing hands , distancing , staying home and have done so since the outset
31	I use all the advised precautions to avoid infections
32	The government put the entire country into quarantine, from which we've not long emerged. It appeared to be the right call as Covid-19 is no longer appearing in the New Zealand population and we can go about living as before.
33	Remain indoors. Not go to work. Remain away from other people at all times.
34	I follow social distancing and wear mask.
35	i followed the guidelines because they were put in place for good reason
36	I've followed the government guidelines precisely. I've been much stricter than the official guidelines and encouraged others to follow them instead of bending the rules and justifying it (e.g. \it's okay because...\")"
37	I try to follow all the instructions the government publishes. In our country, anyone who doesn't abide by those rules risks a fine
38	It is important to follow the rules of Government irrespective of whether i am in agreement with them in this regard. Mainly due to the fact that regulations with regard to

staying at home and keeping your distance etc are very obvious to me and something everyone should have done. (my only issues were the timings with when strategies were implemented and how many lives would have been saved had we reacted sooner)

39 I barely leave home, wear a mask in closed public spaces, avoid social gatherings

40 My government decided to do a partial lockdown - people should stay home (I did for three months). Now people can leave but they have to wear mask every time they are in a public space (for example, train), which I do.

41 my husband is in the high risk group so I am very careful to avoid catching the virus

42 Self isolated myself.

43

I want it to end quickly

44 I spend a lot of time reading news about current events.

45 Have obeyed all the national lock down rules

46 I'm concerned about situation and I'm doing my best

47 I try to do my best to help in this situation. I have stayed at home dince lockdown and I havent met with anyone else from outside my household.

48 We follow the guidelines given

49 i follow the rules protect myself and people who surrounds me

50 I have been following social distancing protocols.

51 To be honest, I have been following these measures. However I've been going on advice from scientists, and was doing so long before the government started lockdown. I have barely left the house since the beginning of March. I have had all my groceries delivered. I have not seen any of my friends or family, except for my partner who I live with, and my father who I occasionally drop shopping to. We've had a couple of conversations at a distance.

The government has now allowed people to meet, and pubs are opening on Saturday which I find ridiculous. I will not be going out to socialise anytime soon.

So yes, I have been following government measures. But I've been doing more than that and will continue to do so.

52 we have observed the lockdown and restrictions

53 My country took good measures, they asked to stay home or wear a mask and wash hands frequently in case you go out.

54 Our Govt is tring to stop this, and is taking all measures they can to stop the deaths and sickness

55 I believe in following the laws of a government, unless it causes me to compromise my moral principles, and also my husband is in a vulnerable group due to heart and blood pressure issues, so I am concerned to keep him safe.

56 I keep an eye on events.

57 I stay home for the last 3 months and do internet shopping. I don't go out as I have a small garden. However I wear mask if I have to go somewhere and keep distance from others.

58 I wear a mask all the time when I am in a place outside my house, I wear hand sanitizer all the time and wash my hand always that I arrive home. I don't see a lot of people at a time and also don't touch people that don't live in the same house as I do.

59 The best source is gis and data, because I can see there actual data

60 Always keep up to date with new developments

61 /

62 i have stayed at home for 107 days so far

63 Because I am in an at risk age group. I don't want to catch the virus or, in doing so, pass it on.

64 It was important to understand everything that was being done and advised.

65	I'm still social distancing, even as services are reopening, the school work I do is all online, I wear a mask when I go to closed spaces, or when I know I might be surrounded by people (market, post office). I havent travelled at all or used public transportation.
66	Since the announcementS, I take great care in preventing the spread. I now wash my hands constantly, wear a face mask when out and always have hand sanitizer.
67	My country had good mesures to the corona virus but i think they implemented it too late. People don't always follow the mesures
68	Most of the time I stay home, I take my dog out once a day to the park but stay away from everyone and come right home
69	I always use masks when it's required
70	I've been following the measures imposed by my government, because it's important to stop the spread of the virus, and we all should do our job to avoid the negative impact of this disease.
71	Washing hands as much as possible, wearing face coverings when outside, avoiding shopping and non essential journeys, no travel for holidays.
72	I follow all the recommendations. I try to keep my social distance and wear a mask in public places. I believe that they are effective methods.
73	follow masking, hygiene, social distancing rules.
74	I have only left the house for essential purchases and I always wear a mask. I have not met up with people outside my household and have limited how much I travel.
75	I haven't left the house since lock down apart from a daily walk close to home. I also do not meet anyone apart from who I live with.
76	i always follow them
77	I have adhered to government advice and have stayed home .
78	It is very important to follow the lockdown measures in order for coronavirus cases to slow down.
79	I have followed advice by staying at home and only going shopping for food

80	I don't think I have ignored anything the government suggested we do.
81	I follow the measures that the government took by staying at home as much as I can and by using a face mask whenever I need to leave my house.
82	They are the people who know what exactly is happening
83	I haven’t been to see any of my relatives since lockdown started, haven’t ordered takeaways, haven’t traveled more then 10 miles and basically only for work and groceries shopping
84	I wear masks when I have to go outside.
85	Have self isolated and social distanced washed hands regularly and only gone in essiential journeys
86	I follow they advice on a daily basis. They ask that we practice social distancing, keep crowds to a minimum and wash your hands often.
87	I am required to wear a mask so I do. Very simple. And I no longer entertain at home.
88	As a key worker i find it important to follow the rules. The quicker we adhere to them the quicker it will be back to â€˜normal’
89	I always adhere to social distancing, I regularly wash my hands and I dont have people in my home
90	I avoid getting out of the house unless I really have to. When I'm out I wear a mask and keep my distance from other people. I'm extra careful when sneezing or coughing and when it comes to washing my hands.
91	We all have to play our part.
92	I do not leave the house unless essential
93	Wearing a mask in public, self-isolating
94	I do everything that the government say's it's correct to do in my country

95	I stay home unless it's essential for me to go out. I always wear a mask while outside and I ensure I prtactice social distnacing.
96	i always wash my hands and use the mask and gloves if are necessary
97	im sticking to the guidelines as much as humanly possible
98	I always wash my hands, use mask and mantain social distancing
99	I stayed home as recommended, I do social distancing and use mask when I have to go shopping and/or to pharmacy and public places.
100	Lock down and social distancing was needed to save this country (Ireland) from thousands beyond thousands of more deaths.
101	I practised social distancing and self-isolation.
102	I have been social distancing, washing my hands, and wearing a mask.
103	When my country was in quarantine I followed the rules.
104	I wash my hands, use gel when out and about and wear a face mask. I stay at home and only leave for essential journeys.
105	I stayed home during lockdown and have been staying home ever since it was lifted. However i now go out more for groceries and meet with a small group of people.
106	follow all rules given
107	stay home, social distance, masks
108	I have to do my part to reduce the spread of the virus and help myself as well.
109	i have always practised social distancing, going out for 1 hour exercise etc
110	My government is a complete clown show. That said, I wear a mask and gloves whenever I leave the house, which is generally once per day to get sunlight and exercise. I social distance. I don't congregate.
111	I have remained in doors other than travelling to work. I have not seen family or friends.

112	I only shop when I have to and I follow all the advice from government when I see it on TV.
113	Always follow as I don't want to be the carrier and spreading it to my loved ones.
114	I am always aware of the new measures, and rules are imposed by my government
115	I try to socially distance when I can. If I do leave my home, I wear a mask.
116	My local government has recommended certain practices from early on in the pandemic like staying at home and wearing a mask, which is still in place.
117	I rarely go out an about and if I do I always wear a mask.
118	I wear a mask when outside and am careful to self-distance. I wash my hands regularly. I avoid going out unless it is absolutely necessary.
119	I stay home often, social distance and wear a mask in public. These are sensible things that can help me, my family and the country as a whole. To do less would simply be irresponsible.
120	We have been under strict quarantine and continue to be more conservative as things open up.
121	as government showing their respons to the cause it can be say as they vvr showing concern always.
122	Since the implementation i have not left the house other than to walk the dog or hanging out in my garden, while complying with social distancing measures. I have avoided coming into close contact (within 2m) of anyone other than people i live with.
123	I followed every measure the Government put in place to slow down the spread of COVID-19 when we were told we had to apply for a permit to go shopping and other necessary activities, I never went out without applying, and I made sure I did not go out unless it was absolutely necessary.
124	I find it important so I follow the rules that are given
125	I try to socially distance when I'm out, I always wear a mask, I practice good hygiene, and try to stay home as much as I can.

126	My husband and I have stayed at home since mid-March. The only place we have gone is the grocery store. We always wear our mask as well.
127	I think it's important to follow the guidelines to reduce the risk of a second wave.
128	I follow the developments of my governments' reactions to COVID-19 closely, on federal, state, and local levels.
129	I always follow Government's initiatives against COVID-19.
130	I wear face mask all the time and I try to avoid going out.
131	I always wear a mask, and try to keep my distance from people at least 6ft.
132	I live in Illinois and I think my state government has handled the pandemic very well. They were very quick to issue lockdowns and have stressed the importance of social distancing from the start. I think they were a great example to follow.
133	I follow the social distancing that was imposed on us. And also wash my hands almost everytime.
134	I follow all the social distancing rules as well as wear the mask and wash my hands regularly and avoid gathering in crowds.
135	I wear a mask indoors, wash hands often and try to keep distances.
136	I just don't meet anyone when it's not necessary, I always put on a mask and gloves in the store. Despite these protections, I wash my hands and hands thoroughly after returning home
137	I wear a mask, keep a 2 meter distance from other people, avoid touching hands, etc. as per the instructions.
138	I am not taking a chance with my life
139	I followed them as I was worried about spreading to my family and their health
140	I don't have really anything better to do.

141	I follow measures to protect people from COVID-19 carefully. I wear a mask in public, have been socially distancing since March, wash hands frequently, and I will continue to follow these measures even as restrictions loosen.
142	I wear the mask and social distance
143	I quarantined at home and followed safety guidelines
144	I socially distance, I stay home if possible, I wear a face covering and I wash my hands.
145	Followed lock down and contact guidance
146	We have been diligent in adhering to distancing and masking protocols. We have made every effort to isolate and only expand our social bubble when appropriate. We wear masks and clean our hands when in public settings, to protect ourselves and others. The only time I don't wear a mask is to walk the dog as I am in the suburbs and can do so without coming into close proximity with others.
147	I follow all the orders such as wearing mask, social distancing, not going out and staying in if I feel unwell.
148	I have stayed at home except for essential food shopping and medication collection
149	I have quarantined, worn face masks when outside, and engaged in social distancing.
150	Wearing mask, social distancing, going out when necessary and staying home as much as possible.
151	I always wear masks when in public places and wash my hands frequently, also I avoid going into crowded places.
152	socially distancing, avoiding uneccessary travel and shopping
153	I have been shielding from the virus for 3 months. working from home and only going out of my house if necessary
154	I am constantly looking for updates on how both the federal and state government are handling the pandemic.
155	I always follow whatever is recommended by the government and RKI.

156	I believe we all need to follow them to prevent the spread
157	Always keeping 2m apart and always washing hands. Sanitising also before entering shops.
158	I follow it because i want to be as safe as possible

Participants who ***often*** follow measures

Reference nr.	Response
159	I'm uneasy at how fast we are attempting to go back to normal. We've just shut an entire city, and yet pubs are re-opening on Saturday. It seems too fast.
160	I practice social distancing and wear a mask when going outside
161	Quarantine. Washing hands and wearing masks.
162	Recently I have become slightly compliacant with the rules. Have not been wearing a mask
163	I follow the government news on the tv about the coronavirus
164	With the current relaxing on the restrictive measures, I feel that I am ofter doing more than the recommended by the government.
165	I wear masks, I'm social distancing and I stayed at home most of the time
166	make new laws, isolation of population, quarenteen
167	I am interested in stopping the spread of the virus so I practice social distancing etc
168	I am often agree with italian government choices about covid because they tryed to save as many lives they can
169	I often wear a mask, especially in my work settings. I always disinfect my hands and wash my hands. However sometimes when I go for a quick trip to the supermarket I don't wear a mask.

170 I am changing countries frequently due to work but overall I follow all rules and try to do my best.

171 I am following government guidelines to stop the spread of virus

172 i practice social distancing, wash my hands frequently etc but not as much as i should

173 I don't use public transportation if possible, staying far from other people. I used to stay at home working according to the advice

174 i wash my hands, wear mask and social distance

175 I follow social distancing & wear a mask if I am told to.

176 I am working from home and only leaving the house for essential reasons, although I have been seeing one or two family members due to health reasons.

177 I complied with all rules and restrictions but I have not changed any of my behaviours since March, by that I mean I am not relaxing my behaviour as much as my government has said I can, as I do not believe it is safe to do so.

178 I am keeping up to date every day with the government advises and I am following them as much as I can

179 It happened a few times that I left home without good reason, but generally I follow the measures because I think they can slow down the virus spread.

180 I am careful everyday. I wash my hands and use a mask

181 if things are closed i will respect that. i will not go out to bars and restaurants if they havent re-opened. i will stay home most likely anyway

182 I tend to stick to the rules of the restrictions, but occasionally 'bend' them slightly to do things that I enjoy doing. I feel most other people are doing this to a much greater extent than i am

183 i often look at the current situation to see what new guidelines are being used

184 I started following what was happening in Italy and Europe and changed my behaviour before the UK Government did anything. When their advice was to wash your hands I was already staying home as if I was under lockdown. Now they are cutting social

distancing from 2m down to 1m and reopening pubs and shops I won't be changing my behaviour. Florida and Texas tried this and have already closed their bars again.

185 I always use a face mask when I go out. Now when it is not mandatory, I wear it during shopping.

186 Because it will depend how i will act during the pandemic situationand know the updated work rules during Covid.

187 I wear protective masks

I disinfect my hands after each use of general equipment

188 I tend to follow recommendations.

189 I believe that this was an unknown virus and no one was prepared. On the whole I think they have tried their best supporting people with furlough scheme etc. They do have questions to answer though

190 When going out in public I try to follow the social distance measures where I can.

191 I try to follow them all the time but it's difficult when I encounter someone else who doesn't and stands too close to me for example.

192 I often try to disinfect my hands. I have to wear a mask at work. As the epidemic grew, I didn't leave the house much.

193 There are not many measurements that the Dutch government took.

- Public transport = use a mask - Ergo, I don't use public transport for a while. I do have masks at home for whenever I need to.

- 1.5 meters apart. = Love this one, but you can't always keep 1.5 meters apart, for example in the store when people just sneak the fuck up on ya. Same on the streets. I can try to keep distance, but the only way to do that is not to go out at all, and I do go out for air and exercise.

194 Generally I agree with measures taken by my GOV but I believe that in some cases they overreacted. In some cases it's simply not possible to follow it.

195 I often obey restrictions.

196	I usually try to keep distance and always wear a mask when traveling with public transport
197	In my country, the measures were not very severe. But I started a teleworking regime and avoided contact with other people as much as possible. I avoided using public transport.
198	I am a key worker therefore I was unable to be in total lockdown
199	I have often followed the measures taken by the local government.
200	I followed most of the rules, although did meet 2 friends at a distance outdoors, at the time we could only meet one person.
201	I wear a mask in stores, I often disinfect my hands
202	I tried to always follow the rules but I sometimes unconsciously forgot to follow the rules, forgetting to wear a mask, for example
203	I have more of less quarantined myself for the entire time and social distance and wear a mask when i wasnt able to stay home. I dont think hand washing does much, it was mostly busywork to make people think they had control over the scary situation. I wear a mask
204	I comply with all the recommendations implemented by the government of my Polish country
205	I follow most measures but not always be possible
206	I practiced social distancing for a long time before getting a job during the coronavirus outbreak. Once I started working I had to start wearing gloves and face masks especially on transport.
207	even a month ago I adhered to rules such as wearing a mask, but after removing this order I do not wear it so often. However, in places where there are a lot of people, I put on a mask.
208	The restrictions and instructions government gave have to be followed.
209	often - not always but more than sometimes

210	Had to self isolate when came back from a work trip and have been on lock down for 3 months
211	I feel the response by the Scottish government has been generally good, more cautious than the UK government but it feels more appropriate given the seriousness of the virus.
212	For the first 6 weeks since lockdown was announced I did not leave my house - I was a lot more worried about getting the virus, another member in my household was responsible for shopping. Since then, I've seen a lot of people on social media meeting with their friends, not adhering to social distancing and I began going outside just to go to supermarkets (this was really exciting and felt like a big deal!) and for walks at the beach alone.
213	Some measures are literally impossible to follow, for example keeping the social distance of 2 meters in narrow streets.
214	I try to wear masks at all timer, sometimes LaTeX gloves as well
215	I do follow them. Nearly always.
216	I try to follow it as often as possible. However, there was a situation that the Polish Prime Minister went to the cafeteria without the face mask and this made me questioning all the measures. Also this was not the only one of such situations.
217	Keeping up to date with release from lockdown
218	Stopped socialising, stayed at home, avoided all unnecessary contact
219	I think they were delayed in the response.
220	During in the pandemic I had to travel back to my university house to remove all the items and clean it thus breaking my countries travel limitations
221	I am currently quarantined and when leaving the house, only for essentials such as a weekly food shop, I remain 2m apart from other shoppers and always wear a mask.
222	Sometimes it's hard to follow 100% specially when government first says one thing is correct/wrong and then a few weeks later changes their mind
223	I try to stay home as long as I can, and always wear a mask, but I've been to a friend's house without a mask.

224	i stay at home most of the time and allways use the mask when i go outside
225	I always wear a mask when I go out and distance myself at least 6 feet from others.
226	I believe their advice to be in my best interests
227	I think about it but I don't always keep enough distance
228	I go above what the rules are, I will not be following the more relaxed rules
229	I am doing what applies to me most of the time. I wash my hands after being out, use hand sanitizer when I can't, wear a mask in public and stay home when it isn't necessary to be out.
230	I keep social distances most of the time and I always wear a mask
231	I try to limit my social contact as much as possible without going too crazy. I social distance when I am around other people
232	Well many people aren't following the rules for social distancing, whether you look at shops here the number of people inside shops are not maintaining social distancing, shops solely are worried about making as much money as they possibly can, what is the point of having a queue outside the shop when inside no one is 2 metres apart. You can only do so much when other people aren't doing the same.
233	i wash my hands often spray antibak around the house, wear mask when near others and try to keep a safe distance from others i also dont go out often
234	Sometimes i would not implement the correct measures when going for a walk or doing menial tasks where i am outside but not in contact with others, such as not wearing a mask.
235	Only because it was ordered to.
236	I try to wear a facemask everytime I come out, but at the beginning, I think that I did not respect all the measures that the government imposed
237	I try to follow all protocols, except when getting groceries or walking the dog.
238	I live inn Germany currently and I agree pretty much completely with how the German government dealt with the situation. However, I think they were too quick to bring football

back...

I am from the UK however, and totally disagree with how the British government have handled things... they waited too long to lock down and not enough has been done to help the healthcare system, and there has never been any rules for wearing masks or anything. The are also opening everything up too soon, when the cases are still high. Nicola Sturgeon, leader of SNP in Scotland, has however handled everything admirably

239	I'm afraid for my loved ones and I follow the rules
240	Im a care worker so have been working right through COVID-19 but in my home and personal lifes we have followed the rule
241	I listen to my local health officials and wear a mask and social distance; I don't listen to the president
242	try to follow guidance but other people chin it off so i give up a little too
243	2 metre social distancing, masks
244	I don't frequently go outside, but when I do, I make sure I protect myself and keep my distance as instructed. While inside, I also take precautions such as constantly washing my hands.
245	i do everything they ask except for wearing a mask.
246	I do everything they asked for to stay safe from the virus and protect others.
247	As much as possible I try to follow the measures implemented.
248	I follow these rules I agree with.<br><br>I do it for my own safety, not for the government.
249	I'm worried about the virus, so I often check up on about it.
250	We live in a society so it is important for everyone to follow the government rules
251	I only go out for essentials. I have my house and pool for the kids so we only go out when we have to. We are not taking any chances. We wear a mask when we do go out, and if we think the store is too crowded, be just wait until it clears out.

253 I try to follow (as bes that I can) the regulations and recommendations that the local, provincial and federal governments put in place to stop the spread.

254 Face masks in areas with large gatherings - always

Staying inside if not necessary - sometimes

255 my government told me to wear mask and practice social distancing. I follow my government

256 I'm not perfect, but I try very hard to follow the guidelines and am successful at doing so about 95% of the time. Occasionally I just have to go out to get something that might not be considered essential and I like to run outside. I've also forgotten my mask once or twice.

257 I use mask in public places and I try to keep social distance

258 I social distanced from everyone for 17 days. I wear a mask in the stores and I keep the 2m distance too.

259 I sometimes forget to wash my hands but I do try to socially distance and use a mask etc.

260 I follow the guidelines although I may not agree with them unless I consider to follow them would be detrimental to my or my family's health. For example, I continued to visit my 82 year old mother every day despite it not being technically allowed as I considered it more important for her health that she had company than that she stayed at home alone. I also encouraged her to go out to collect her newspaper and do her own shopping despite her being 82 as she is fit and healthy and I thought it better for her physical and mental health to do so.

261 I follow the guidelines as much as I possibly can. I wash my hands or use hand gel often and adhere to social distancing when out and at work

262 i do my best to social distance and wear a mask

263 I follow them to prevent myself and family becoming I'll

264 I social distance and use masks

265 I always use masks, if I feel sick, I self-quarantine, I social distance in a social place.

266	I'm constantly checking current restrictions
267	Social distancing, wearing a mask
268	I followed most of the rules and was mostly careful
269	I always wear a mask and I use hand sanitizer when I go out and do stuff. I don't go out and do much of anything.
270	I try to keep all the guidelines in order to increase the safety of myself and those around me. Sometimes, however, this is not possible when, for example, I would have to wear a face mask all day at work, even when I do not meet other people. I believe that the best combination is to follow the organisation's health guidelines and common sense.
271	I mostly stay at home and avoid going out, sometimes I hang out with 2 or 3 friends, we live in an rural area where there are no cases.
272	I avoid public places, wear masks on transport, stay at two metres distance from people I don't live with.
273	I don't always wear a mask when I go out, if I know that I won't be interacting with others.
274	Sometimes I forgot mask or gloves
275	I read information in the media describing measures prepared by the government.
276	I stayed home as much as possible; I always wear a mask when I do go out; I've taken a few walks around the city centre which, while they don't violate any rules, arguably violate the spirit of the rules, since I was not walking to a specific place for a specific reason. I'd argue it was in great benefit to my mental and physical health however.
277	I socially distance when possible, and I wear a mask when I go out in public.
278	I stay home unless I absolutely have to be out, and I try to always wear a mask when in public, though rarely I won't have one with me.
279	When Shopping I always follow the given rules but sometimes I do not wash my hands

Participants who ***sometimes*** follow measures

Reference nr.	Response
280	I think the Government did not act really well
281	I think they could have acted quicker, but they are trying now and it is a hard task.
282	I read what measures is/was taken if they show up while browsing.
283	It's enough for me to follow these measures only sometimes, because I'm a university student and I've been able to stay at home and study from home without any issues so far.
284	I look less and less at information related to the virus, but I try to know about important rules, for example that I have to wear a mask in a tram or a shop.
285	Not exactly believing in the whole virus, it seems to be exaggerated, although people die, but they died and without the virus more people die from the flu.
286	I don't really watch TV, so from time to time I update myself about it, and also my family keeps me up to date.
287	I used to follow the news initially then I got news fatigue hearing about it all the time
288	I do not believe that virus exist in the dimensions they say, I comply with basic hygiene rules such as washing my hands and having a respiratory label, but I do not stay at home isolated or miss my family
289	have to cue at shops, not through choice. cant go anywhere cos nowhere open.
290	There were some decisions that are taken on the base of fear and population control, and although is necessary i don't like it.<br><br>And also felt like our liberty was being paused.
291	Because some measures were not clear
292	The environment that I work in makes it difficult to socially distance. The rules for this seem to have been disregarded

293 I wear mask and practise social distancing.

294 I have gone against the guidelines and visited friends and family inside the home

295 I read about it in news websites.

296 I FEEL THAT MY GOVERNMENT AS SUCH NEVER TOOK THE EXACT MEASURES TO TREAT COVID-19.

DID NOT HAVE THE RESOURCES TO DEAL WITH THE SITUATION.

297 I try not to go out and when I do I stay with people I’ve already been around

298 i stopped watching press conferences after a few days. so much conflicting information

299 I'm trying to be up to date, but actually in area i live in there is end of epidemic so i just want to know what government is doing as i don't trust them

300 I would not necessarily search or look up the information, but if it is mentioned in an article or programme, I would listen and acquired said information.

301 I have COVID-19 fatigue. The peak dates were wrong for my state. The virus is surging and we haven't hit our normal flu season yet. I feel the government is impotent in this fight because they will not impose strict rules. Just mandating masks would help immensely. The whole situation is so depressing I cannot immerse myself in the data.

302 I have watched with dismay the lack of reaction that our federal government has had. The state government was faster, but still waited until things were pretty much out of control. My doctor has been watching all of it and knows what care I need. I trust him the most.

303 I try to avoid places with many people, I wash my hands regularly and try to avoid touching my face but I don't usually wear a mask when I am in a public place.

304 my government not always align with the health care official guidelines. I \follow\" the measure of my government only when coincide with the official health organization guidelines."

305 If referring to our federal government, I don't feel like there have been explicit guidance coming from the top. So, I try to follow what I see and hear from medical sources and from other countries that appear to have successfully contained the virus for the moment.

306	While I don't practice social distancing as much as I should, I do wear a mask when out in public. However, that is only because they made it mandatory. If masks were still optional, I probably wouldn't wear one.
307	I try to keep practicing social distancing even though my government doesn't demand it
308	I wear a mask when shopping and in a larger group of people. I try to avoid contact with other people, especially those who came back from abroad
309	I don't wear a mask but I do practice social distancing
310	I don't trust my government, it is not trustworthy source.
311	I don't trust my government (I'm from Poland), I prefer to get any knowledge about Covid-19 from international sources.

Participants who ***rarely*** follow measures

Reference nr.	Response
312	The government didn't really seem like they knew what they were doing at the start. Measures were implemented in the course of the past few months that were more totalitarian than to do with virus prevention.
313	They are not good at it
314	Not enough
315	Lockdown was too late, message to mixed, lack of PPE. And Dominic Cummings!
316	the government have shown their incompetence. I follow sensible, common, sense.
317	i don't think action was taken fast enough in forcing lockdown laws
318	I just don't want to take part in it, it's all utter nonsense and lies, everything. Some things I have to do because we all need food, but I will avoid queues, masks, public transport (because of masks),tests, apps, digital passports and especially vaccines. I'm Out!

319	Eveyone wear mask here not covering their nose so wearing it or not doesn't make a difference. Moreover, I believe masks are made to be worn in order to destroy humans natural bacteria flora which strenghthen itself by having facial and hand contact with others.
320	Our government are a shambles

Participants who ***never*** follow measures

Reference nr.	Response
321	There is no covid-19
322	I was social distancing two weeks before the government called for lockdown and I will stay distancing until we know more. The government seem less concerned with health than with economy and 'herd immunity' even now.
323	My government did the exact opposite of what every health organization said to do. I'm not following them off a cliff
324	The 6 foot rule is a joke. I don't where a mask because I know for sure I am not sick, and wearing one would not protect me from those who are sick and are not wearing a mask.
325	They opened up clubs and bars in the middle of this, theyre fucking idiots
326	I don't watch the news or listen to it in the radio. I think its conspiracy
